# PLIN2 Promotes Lipid Accumulation in Ascites‐Associated Macrophages and Ovarian Cancer Progression by HIF1α/SPP1 Signaling

**DOI:** 10.1002/advs.202411314

**Published:** 2025-02-07

**Authors:** Hui Luo, Xiaolu She, Yubo Zhang, Bingfan Xie, Shibo Zhang, Qianqian Li, Yangyang Zhou, Shuang Guo, Shushan Zhang, Yanhui Jiang, Yingying Dong, Jianzhong He, Lijie Wang, Qianqian Zhang, Yuan Zhuang, Panxia Deng, Feng Wang, Jihong Liu, Xin Chen, Huilong Nie, Huanhuan He

**Affiliations:** ^1^ Guangdong Provincial Engineering Research Center of Molecular Imaging Guangdong‐Hong Kong‐Macao University Joint Laboratory of Interventional Medicine The Fifth Affiliated Hospital of Sun Yat‐sen University Zhuhai Guangdong 519000 China; ^2^ Department of Gynecology The Fifth Affiliated Hospital of Sun Yat‐sen University Zhuhai Guangdong 519000 China; ^3^ Department of Ultrasound The Fifth Affiliated Hospital of Sun Yat‐sen University Zhuhai Guangdong 519000 China; ^4^ Cancer Center The Fifth Affiliated Hospital of Sun Yat‐sen University Zhuhai Guangdong 519000 China; ^5^ Department of Gynecology Oncology State Key Laboratory of Oncology in South China Sun Yat‐Sen University Cancer Center Guangzhou Guangdong 510060 China; ^6^ State Key Laboratory of Quality Research in Chinese Medicine Institute of Chinese Medical Sciences University of Macau Macau 999078 China

**Keywords:** ascites, hypoxia, macrophages, ovarian cancer, PLIN2

## Abstract

A major characteristic of ovarian cancer (OC) is its unique route of metastasis via ascites. The immune microenvironment in ascites remains understudied, leaving the mechanism of ascites‐mediated abdominal metastasis obscure. Here, a single‐cell transcriptomic landscape of CD45^+^ immune cells across multiple anatomical sites is depicted, including primary tumors, metastatic lesions, and ascites, from patients diagnosed with high‐grade serous ovarian carcinoma (HGSOC). A novel subset of perilipin 2 high (PLIN2^hi^) macrophages are identified that are enriched in ascites and positively correlated with OC progression, hence being designated as “ascites‐associated macrophages (AAMs)”. AAMs are lipid‐loaded with overexpression of the lipid droplet protein PLIN2. Overexpression or suppression of PLIN2 can enhance or inhibit tumor cell migration, invasion, and vascular permeability in vitro, which is also confirmed in vivo. Mechanistically, it is demonstrated that PLIN2 boosts HIF1α/SPP1 signaling in macrophages, thereby exerting pro‐tumor functions. Finally, a PLIN2‐targeting liposome is designed to efficiently suppress ascites production and tumor metastasis. Taken together, this work provides a comprehensive characterization of the cancer‐promoting function and lipid‐rich property of ascites‐enriched PLIN2^hi^ macrophages, establishes a link between lipid metabolism and hypoxia within the context of the ascites microenvironment, and elucidates the pivotal role of ascites in trans‐coelomic metastasis of OC.

## Graphical abstract

This study interrogated the single‐cell transcriptome of immune cells across multiple anatomical sites in ovarian cancer patients and identified a novel subset of macrophages that are enriched in ascites and overexpressed the lipid droplet protein PLIN2. These macrophages promote ascite formation and cancer metastasis through the HIF1α/SPP1 pathway. Treatment with newly developed PLIN2‐targeted liposomes is able to harness disease progression.

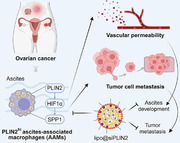

## 1 Introduction

Ovarian cancer (OC) is the most lethal gynecologic malignancy. High‐grade serous ovarian carcinoma (HGSOC) represents the most prevalent and aggressive subtype.^[^ ^1^ ^]^ Patients with HGSOC often present with advanced disease, and one‐third of them carry ascites at the time of diagnosis.^[^ ^2^ ^]^ Malignant ascites not only impair patient quality of life but also contribute to mortality,^[^ ^3^ ^]^ thus becoming an unfavorable prognostic indicator. Moreover, ascites have long been viewed as an indispensable medium, even a prerequisite, for the sinister process of trans‐coelomic metastasis, which ultimately leads to recurrence and poor clinical outcomes.^[^ ^4^ ^]^ Nevertheless, the underlying pathogenesis of malignant ascites is intricate, and the mechanisms by which ascites facilitate transcoelomic dissemination remain elusive, rendering current treatment modalities inadequate.

Immune cells constitute a critical proportion of the tumor microenvironment (TME).^[^ ^5^ ^]^ Immunotherapy targeting the TME has yielded promising outcomes, yet only a minority of patients respond to such treatment due to the complexity and heterogeneity of the TME.^[^ ^6^ ^]^ The immune microenvironment in malignant ascites can exhibit notable distinctions compared to its solid tumor counterpart.^[^ ^7^ ^]^ As a prominent immune cell type in the immune microenvironment, tumor‐associated macrophages (TAMs) master immune regulation by suppressing inflammatory responses, thus fostering cancer progression.^[^ ^8^ ^]^ In ovarian cancer, TAMs have been documented for their pro‐tumor role through the release of various factors, including cytokines, chemokines, enzymes, and exosomes, which directly enhance pro‐survival signaling. Notably, there are some other functions of macrophages in ascites such as facilitating spheroid formation and cancer cell adhesion.^[^ ^9^ ^]^ Despite the comprehensive characterization of the immune landscape in numerous cancers at the single‐cell level,^[^ ^10^ ^]^ how the heterogeneity and function of ascites macrophages contribute to this deadly disease remains to be fully elucidated.

The lipid‐enriched microenvironment in ascites not only provides an energy source for ovarian cancer cells to metastasize^[^ ^11^ ^]^ but also primes the metabolism of immune cells, particularly macrophages, to become a pro‐tumorigenic niche.^[^ ^12^ ^]^ Lipid‐associated macrophages (LAMs) have been implicated in promoting cancer progression and metastasis by manipulating the hypoxic tumor microenvironment.^[^ ^13^ ^]^ Perilipin 2 (PLIN2), a member of the perilipin protein family, is uniquely expressed on the surface of lipid droplets (LDs) that are involved in phenotypic modulation of immune cells, such as macrophage polarization, which culminates in tumor progression.^[^ ^14^ ^]^ Therefore, the elevation of PLIN2 is considered a marker for LAMs.^[^ ^15^ ^]^ However, the precise role of PLIN2 and the downstream mechanism of LAM‐mediated progression and metastasis of OC remain unclear.

The TME is characterized by a state of hypoxia, while ascites worsens tissue's access to oxygen, resulting in an exacerbated hypoxic environment.^[^ ^16^ ^]^ To combat this, cancer cells have evolved to employ versatile mechanisms to respond to hypoxia, including the promotion of angiogenesis to alleviate oxygen stress through aerobic glycolysis.^[^ ^17^ ^]^ In addition, cells within the TME, such as macrophages, have adapted to limited oxygen availability by regulating glycolytic metabolism, redox homeostasis, and inflammatory responses.^[^ ^18^ ^]^ Of note, LDs play a critical role in the adaptation of macrophages to hypoxic stress due to their documented functions in lipid metabolism through hypoxia‐inducible lipid droplet associated (HILPDA).^[^ ^19^ ^]^ PLIN2, a marker of macrophage LDs, is essential for cell survival under hypoxia and its expression can be stimulated by HIF1α in post‐ischemic brain injury.^[^ ^20^ ^]^ However, the relationship between hypoxia and LDs requires further investigation. Whether and how hypoxia and lipid metabolism are orchestrated in macrophages in the context of TME awaits further investigation.

In this study, we identified a novel subset of PLIN2^hi^ macrophages enriched in the ascites of HGSOC patients and unveiled their pro‐metastatic and pro‐permeability functions in ascites development. We established a link between the lipid‐enriched microenvironment and hypoxia in the setting of malignant ascites, providing additional evidence in support of targeting the ascites microenvironment as a novel immunotherapy in the treatment of OC.

## 2 Results

### 2.1 Immune Landscape of Multiple Lesions in HGSOC at Single‐Cell Resolution

**Figure 1:**
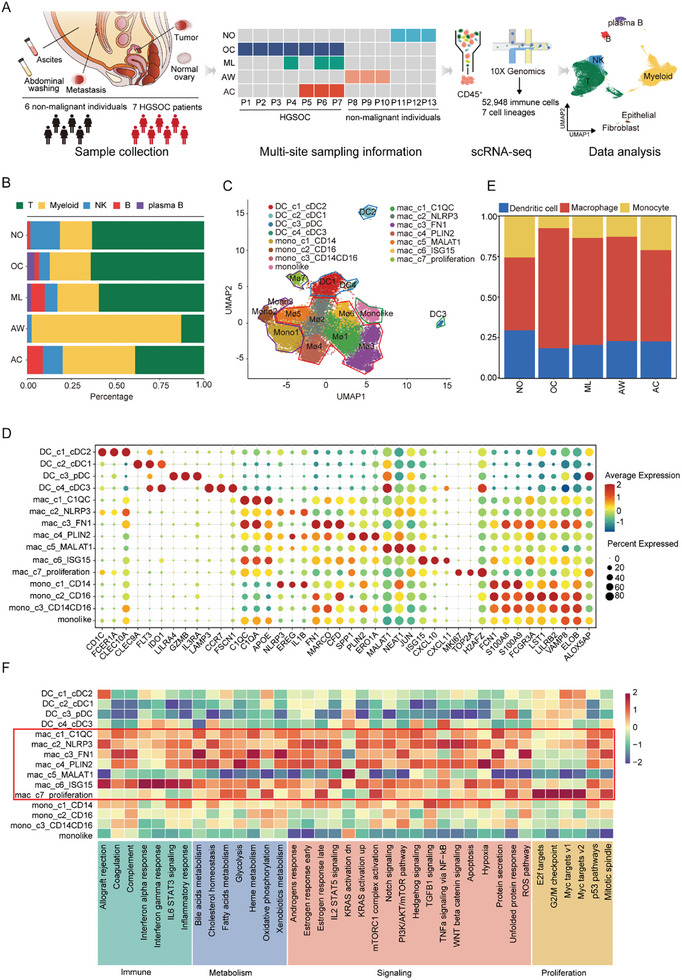
Immune landscape of multiple lesions in HGSOC. A) Schematic design of the study showing the collection and processing of tissue samples from six non‐malignant and seven OC patients for scRNA‐seq. B) Stacked bar chart displaying the proportion of immune cells in different lesions. C) UMAP plot showing 15 clusters of 19336 myeloid cells indicated by color. D) Bubble plot of the top three differentially expressed genes in comparison with the myeloid subclusters in OC. Bubble size represents the percentage of cells expressing the gene in a subpopulation. The color of the circle represents the average expression level of the gene in a subpopulation (from blue to red). E) Stacked bar chart displaying the proportion of myeloid clusters in different lesions. F) Heatmap of hallmark pathways with the enrichment score by GSVA.

To comprehend the ascites‐specific immune microenvironment in HGSOC, we carried out immune profiling of multiple lesions and corresponding control tissues from patients using single‐cell RNA sequencing. A total of 19 samples were obtained from seven treatment‐naïve patients diagnosed with HGSOC and six patients with hysteromyoma or high‐grade cervical squamous intraepithelial neoplasia as non‐malignant controls (**Figure** **1**A and Table S1, Supporting Information). Of note, four sets of matched samples, each comprising at least two different lesions from a single individual, were utilized to further depict the heterogeneity of the TME. CD45^+^ immune cells were harvested by fluorescence‐activated cell sorting and analyzed. Following quality filtering and doublet removal, single‐cell transcriptome data was obtained from a total of 52948 immune cells isolated from the following: non‐malignant ovary (NO), primary tumor (OC), metastatic lesion (ML), ascites (AC), and abdominal washing from non‐malignant peritoneal cavity (AW; Figure S1A, Table S2, Supporting Information).

First, we performed pseudo‐bulk principal component analysis to evaluate the similarity among various lesions (Figure S1B, Supporting Information). A notable finding was the distinction between solid and fluid tissues, which was consistent with the result of Pearson's correlation analysis of the gene expression profile (Figure S1C, Supporting Information). A unique set of highly expressed genes was identified in samples from each site (Figure S1D). Next, we employed the Harmony algorithm to minimize the batch effect of the transcriptomic data from these 19 samples (Figure S1E,F, Supporting Information). To define the cellular composition of these samples, we performed unsupervised graph‐based clustering and differential gene expression analysis (Table S3, Supporting Information). We then annotated major cell clusters by the average expression of curated gene sets (Figure S1G, Supporting Information). Furthermore, we evaluated the distributions of cell types in different samples. Generally, myeloid cells were found to be more prevalent in fluid tissues compared to solid tissues, and the composition of immune cell types exhibited greater heterogeneity in malignant tissues than in non‐malignant tissues (Figure 1B; Figure S1H, Supporting Information). These data depict a distinct immune microenvironment in different milieus.

Since myeloid cells dominate the immune population in both AC and AW, we further analyzed these cells by grouping them into three major lineages, comprising four subclusters of dendritic cells, seven subclusters of macrophages and four subclusters of monocytes based on canonical marker genes (Figure 1C,D and Table S4, Supporting Information). Consistent with previous reports,^[^ ^21^ ^]^ macrophages constituted the most predominant component of the myeloid lineage across all the tissues examined (Figure 1E; Figure S1I, Supporting Information). We then performed Gene Set Variation Analysis (GSVA) using hallmark gene sets^[^ ^22^ ^]^ to reveal the pathway activities in these subclusters. Intriguingly, macrophage clusters showed overall elevated activities in comparison to dendritic cells and monocytes, in line with the previously reported phenotype^[^ ^23^ ^]^ (Figure 1F). These results highlight the importance of macrophages in shaping the TME of OC.

### 2.2 Identification of a Novel Macrophage Subset Enriched in Malignant Ascites

**Figure 2:**
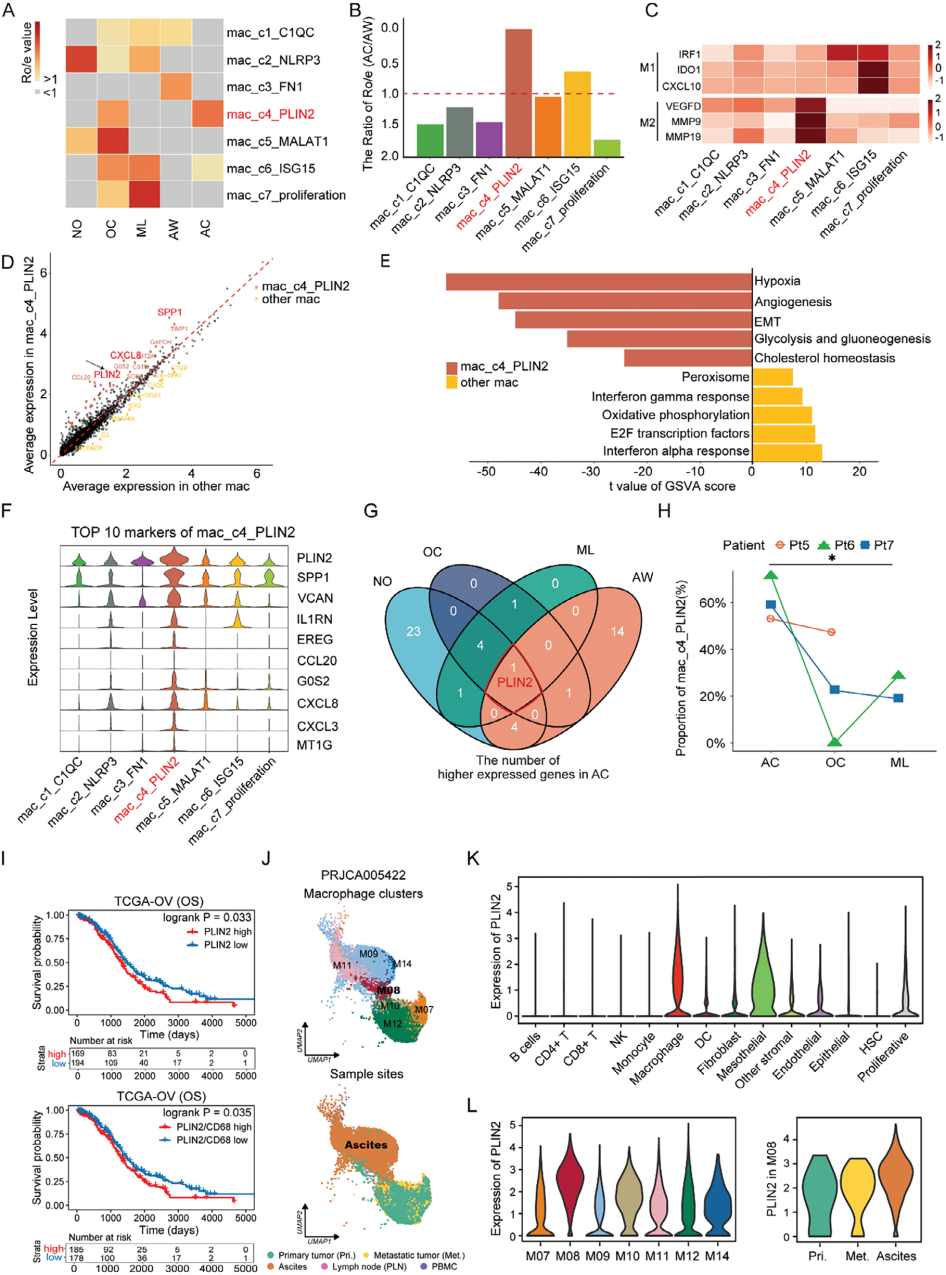
Elevated PLIN2 expression in ascites‐associated macrophages. A) Heatmap illustrating the tissue preference of macrophage clusters estimated by Ro/e value. B) Grouped bar chart specifying the ratio of cell types in different sampling locations. C) Heatmap showing the score of signatures of the classical cell type M1 and M2. D) Scatterplot highlighting the differentially expressed genes between mac_c4_PLIN2 and all other mac subclusters. The x and y axes represent the average expression level of differentially expressed genes in the mac_c4_PLIN2 and other macrophage subclusters respectively. Each dot represents a gene. The color annotation inside denotes an individual gene passing the p‐value and fold difference thresholds; black, non‐significant. E) Bar plot displaying the up‐regulated pathways in both mac_c4_PLIN2 and mac_c6_ISG15 by GSVA. F) Stacked violin plot showing the top 10 marker genes of mac_c4_PLIN2. G) Venn diagram exhibiting the common differential gene with high expression in ascites macrophages compared with the other four lesions. H) Line chart displaying the proportion of macrophages in different lesions of the same patient. I) Kaplan‐Meier plots illustrating overall survival (OS) in patients with ovarian cancer in TCGA stratified by PLIN2 expression or PLIN2/CD68 expression. The cutoff point was determined by maximizing the Log‐rank test p‐value between survival curves. Statistics tables were attached below the survival curves. J) UMAP plot exhibiting the distinct macrophage clusters and sample sites of the PRJCA005422 cohort. K) Violin plots showing the expression levels of PLIN2 in different cell types. L) Violin plots showing the expression levels of PLIN2 in seven clusters of macrophages and three different sites of M08 macrophages. * indicates *P* < 0.05. Paired Student's t‐test for H, log‐rank test for I.

To interrogate the tissue preference for different myeloid clusters, we used the ratio of observed to expected cell numbers (*R_o/e_*) of each subset, as previously described,^[^ ^24^ ^]^ to quantify site‐enriched macrophage subsets (**Figure** **2**A; Figure S2A,B, Supporting Information). Intriguingly, mac_c6_ISG15 was enriched in all malignant solid tissues, including OC, ML, and AC. Unexpectedly, we found a novel macrophage subset, mac_c4_PLIN2, as being most differentially enriched in ascites compared to AW, hence designated “ascites‐associated macrophages” or AAMs (Figure 2B).

The polarization states of macrophages can be quite complex, with the two extremes being classically activated (M1) and alternatively activated (M2). Our data demonstrated that M2‐polarized macrophages constituted ≈50%–70% of all the macrophages in solid tissues, exhibiting a marginally higher prevalence in malignant tissues (Figure S2C, Table S5, Supporting Information). Next, we examined the polarization status of the seven macrophage subclusters identified in our dataset and found that the majority of these subclusters had a mixed polarization profile (Figure 2C). Of note, the AAMs were more skewed toward M2 polarization (Figure S2D, Supporting Information). Considering that AAMs were enriched in ascites and mostly M2‐polarized, we suspected that they might function similarly to TAMs to promote cancer progression in ascites. Indeed, upon meticulous examination of the differentially expressed genes (DEGs), we found that AAMs overexpressed SPP1 and CXCL8 (Figure 2D), which have been widely reported to facilitate tumor progression and metastasis.^[^ ^25^ ^]^ Subsequently, we implemented a GSVA analysis, which led to the identification of significant enrichment of hypoxia and angiogenesis pathways within AAMs (Figure 2E). These results demonstrate that AAMs are skewed toward the M2 phenotype and may promote tumor progression.

To identify the signature gene of AAMs, we analyzed the top ten DEGs in mac_c4_PLIN2, among which PLIN2 was most highly expressed (Figure 2F). When comparing the highly expressed genes of macrophages in ascites with those in other lesions or non‐malignant tissues, PLIN2 stood out as the only specific and highly expressed gene that distinguishes AAMs (Figure 2G; Figure S3A–E, Supporting Information). Furthermore, we used the data from patients with multiple matched lesions to validate the enrichment of AAMs and PLIN2 expression in ascites (Figure 2H; Figure S3F, Supporting Information). Importantly, high PLIN2 expression levels were capable of predicting a poor survival rate among patients with OC (Figure 2I; Figure S3G, Supporting Information). Additionally, we validated that PLIN2 was predominantly expressed in ascites macrophages in another previously published dataset^[^ ^2a^ ^]^ (Figure 2J–L; Figure S3H,I, Supporting Information).

### 2.3 PLIN2^hi^ AAMs Promote OC Metastasis and Ascites Development

**Figure 3:**
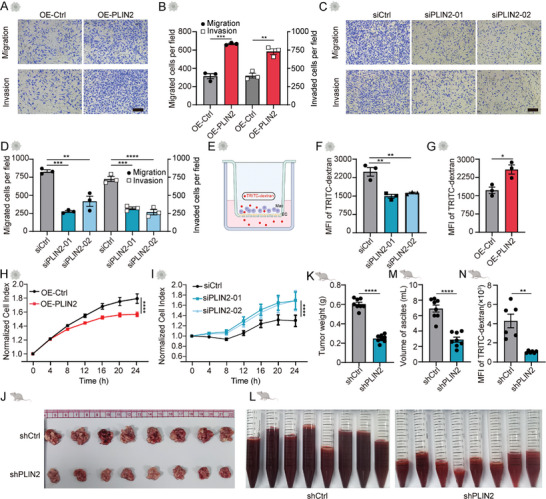
PLIN2^hi^ macrophages promote transcoelomic dissemination and ascites production. A) Representative graph of migration and invasion of SK‐OV‐3 indirectly cocultured with THP‐1 macrophages with (OE‐PLIN2) or without (OE‐Ctrl) PLIN2 overexpression (n = 3). The scale bar represents 200 µm. B) Bar graph showing the statistical analysis of (A). C) Representative graph of migration and invasion of SK‐OV‐3 indirectly cocultured with THP‐1 macrophages that were transiently transfected with PLIN2‐specific siRNA (siPLIN2) or control siRNA (siCtrl; n = 3). The scale bar represents 200 µm. D) Bar graph showing the statistical analysis of (C). E) Schematic diagram of TRITC‐dextran dye extravasation assay. F, G) Quantification of TRITC‐dextran tracer fluorescence from the coculture system with endothelial cells and macrophages that overexpress PLIN2 (OE‐PLIN2) or vector (OE‐Ctrl) (n = 3; F) or are transiently transfected with PLIN2‐specific siRNA (siPLIN2) or control siRNA (siCtrl; n = 3; G). H) Evaluation of cellular permeability in real time with (OE‐PLIN2) or without (OE‐Ctrl) PLIN2 overexpression using the automated system (RTCA; n = 3). I) Evaluation of cellular permeability in real time with PLIN2 siPLIN2 (siPLIN2) or control siRNA (siCtrl) using RTCA (n = 3). J) Representative images of tumors from mice inoculated with ID8 tumor cells mixed with macrophages that are stably knocked down with PLIN2 (shPLIN2) or control cells (shCtrl; n = 8). K) Bar graph showing the statistical analysis of (J). L) Representative image of ascites from mice in (J). M) Bar graph showing the statistical analysis of (L). N) Bar graph showing the statistical analysis of vascular permeability in the tumor model by measuring the leakage of TRITC‐dextran into the peritoneal cavity. Data are presented as mean ± SEM. ^*^ indicates *P* < 0.05, ^**^ indicates *P* < 0.01, ^***^ indicates *P* < 0.001 and ^****^ indicates *P* < 0.0001. Unpaired Student's t‐test for B, F, K, M, N; one‐way ANOVA analysis for D, G; two‐way ANOVA analysis for H and I.

Next, we sought to investigate the functions of PLIN2^hi^ AAMs in ovarian cancer. Firstly, we ectopically overexpressed PLIN2 in THP‐1‐derived macrophages and subsequently incubated the conditioned medium from these cells with human ovarian cancer cells SK‐OV‐3 and OVCAR‐3. The results demonstrated that the migration and invasion of ovarian cancer cells were enhanced by macrophages overexpressing PLIN2 (**Figure** **3**A,B; Figure S4A–C, Supporting Information). Conversely, the migration and invasion of cancer cells were diminished by macrophages with PLIN2 knockdown (Figure 3C,D; Figure S4D–J, Supporting Information). These results were also validated using bone marrow‐derived macrophages (BMDMs) (Figure S4K–N, Supporting Information). Given our prior demonstration of the association between TAMs and vascular permeability,^[^ ^26^ ^]^ we hypothesized that PLIN2^hi^ AAMs could affect vascular permeability. Through the permeability assay (Figure 3E), we observed that a significantly lower amount of dextran traversed when PLIN2‐knockdown macrophages were directly co‐cultured with endothelial cells (Figure 3F; Figure S4O,P, Supporting Information). In contrast, macrophages overexpressing PLIN2 augmented vascular permeability (Figure 3G; Figure S4Q, Supporting Information). Such alterations were also confirmed using electrical resistance analysis (Figure 3H,I).

To further elucidate the role of PLIN2 in ascites development and metastasis, we peritoneally injected ID8 cells mixed with murine macrophages with differential expression of PLIN2. Consistent with the in vitro results, a decrease in tumor size and weight was observed in the shPLIN2 group in comparison to the shCtrl group (Figure 3J,K; Figure S5A, Supporting Information). Critically, suppression of PLIN2 led to significant attenuation in vascular permeability and ascites development when compared to the control group (Figure 3L–N). Complementing this, overexpression of PLIN2 in macrophages promoted metastasis and ascite development (Figure S5B–F, Supporting Information). Additionally, to genetically resemble HGSOC patients, such as p53 mutation, we constructed a peritoneally metastatic ovarian model using HM1 cells in a mixture with RAW264.7 cells with differential expression of PLIN2. The results demonstrated that PLIN2 suppression caused decreased permeability in mouse models, leading to  less ascites and tumor weight (Figure S5G,H, Supporting Information). The above results indicate that the expression of PLIN2 in macrophages plays a pivotal role in the development of ascites and tumor metastasis, both in vitro and in vivo.

### 2.4 PLIN2 Levels Positively Correlate with Lipid Droplet Accumulation

**Figure 4:**
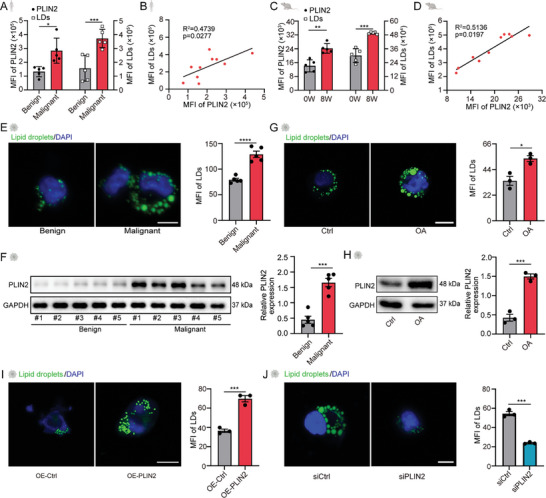
PLIN2 levels positively correlate with lipid droplet accumulation. A) Bar graph showing statistical analysis of PLIN2 expression and LD accumulation in macrophages from ascites of patients diagnosed with HGSOC (indicated as malignant) or benign diseases by flow cytometry (n = 5). B) Correlation analysis of PLIN2 expression and LD accumulation from (A). C) Bar graph showing statistical analysis of PLIN2 expression and LD accumulation in ascites macrophages from ovarian cancer‐bearing mice (n = 5). D) Correlation analysis of PLIN2 expression and LD accumulation from (C). E) Representative images and statistical analysis of LD accumulation in THP‐1 macrophages after incubation with ascites from benign or malignant patients detected by confocal microscopy (n = 3). LDs in green; nuclei in blue. F) Western blot and statistical analysis showing expression of PLIN2 in THP‐1 macrophages after incubation with ascites from benign or malignant patients. GAPDH was used as a loading control (n = 5). G) Representative images and statistical analysis of LD accumulation in THP‐1 macrophages after incubation with OA or BSA (Ctrl) detected by confocal microscopy (n = 3). LDs in green; nuclei in blue. H) Western blot and statistical analysis showing expression of PLIN2 in THP‐1 macrophages after incubation with OA or BSA (Ctrl). GAPDH was used as a loading control (n = 3). I) Representative images and statistical analysis of LD accumulation in THP‐1 macrophages that were harboring PLIN2 overexpression (OE‐PLIN2) or vector (OE‐Ctrl) detected by confocal microscopy (n = 3). LDs in green; nuclei in blue. J) Representative images and statistical analysis of LDs accumulation in THP‐1 macrophages transiently transfected with siRNA targeting PLIN2 (siPLIN2) or scramble siRNA (siCtrl) detected by confocal microscopy (n = 3). LDs in green; nuclei in blue. The scale bars represent 20 µm. Data are presented as mean ± SEM. ^*^ *P* < 0.05, ^**^ *P* < 0.01, ^***^ *P* < 0.001 and ^****^ *P* < 0.0001. Unpaired Student's t‐test for A, C, E‐J. Pearson correlation analysis for B and D.

PLIN2 is a constitutive LD protein. To explore the possible link between LDs and PLIN2^hi^ AAMs, we first attempted to probe PLIN2 expression and LDs in ascites from HGSOC patients. The results revealed that the number of LDs in the ascites from patients with HGSOC was significantly higher than that in patients with benign diseases, accompanied by a concomitant increase in PLIN2 expression (**Figure** **4**A,B). Such alteration was also observed in the ID8 murine model (Figure 4C,D). Next, we sought to identify the source of LDs and determine whether the microenvironment of ascites could induce LDs or spur PLIN2 expression in macrophages. By incubating THP‐1 macrophages with ascites from HGSOC vs benign patients, we demonstrated that malignant ascites, but not benign ones, induced the accumulation of LDs (Figure 4E; Figure S6A, Supporting Information). Meanwhile, the protein of PLIN2 was also elevated (Figure 4F; Figure S6B,C, Supporting Information). To further support our findings, we used oleic acid (OA), a widely accepted reagent for the induction of LDs, to validate the relationship between PLIN2 and LDs. As expected, OA triggered a substantial upregulation of LDs in macrophages (Figure 4G; Figure S6D–F, Supporting Information), accompanied by elevated PLIN2 levels (Figure 4H; Figure S6G–I, Supporting Information). Intriguingly, manipulating PLIN2 levels by either overexpressing or knocking down the protein resulted in a boost or hindrance, respectively, to the accumulation of LDs in THP‐1 macrophages or BMDMs (Figure 4I,J; Figure S6J–L, Supporting Information). Taken together, these results indicate that PLIN2^hi^ AAMs are loaded with LDs, which are likely induced by the lipid‐rich microenvironment in malignant ascites.

### 2.5 PLIN2^hi^ AAMs Promote OC Progression through HIF1α/SPP1 Signaling

**Figure 5:**
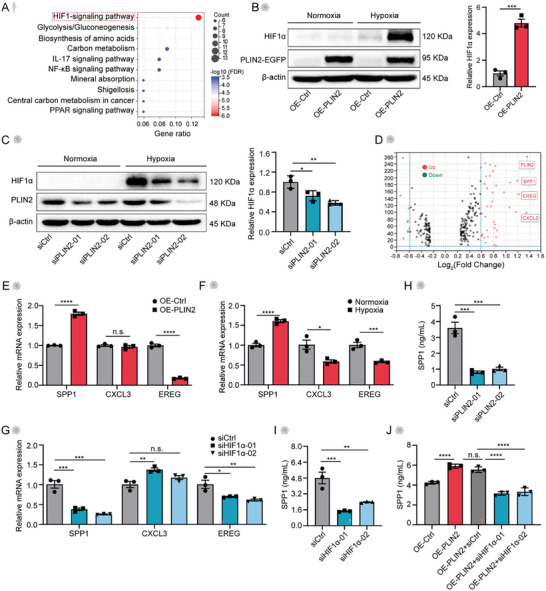
PLIN2 promotes HIF1α/SPP1 signaling in macrophages. A) KEGG analysis of differentially expressed genes (DEGs) of PLIN2^hi^ AAMs. B) Representative images and statistical analysis of HIF1α expression upon PLIN2 overexpression (OE‐PLIN2) or control (OE‐Ctrl) under normoxia and hypoxia (1% O_2_) in THP‐1 cells by Western blot. β‐actin was used as a loading control (n = 3). C) Representative images and statistical analysis of HIF1α expression upon PLIN2 knockdown (siPLIN2) or control (siCtrl) under normoxia and hypoxia (1% O_2_) in THP‐1 cells by Western blot. β‐actin was used as a loading control (n = 3). D) Volcano map of DEGs of PLIN2^hi^ AAMs. E) Representative bar graph showing relative RNA expression of SPP1, CXCL3, and EREG in THP‐1 cells upon PLIN2 overexpression (OE‐PLIN2) or control (OE‐Ctrl) by qPCR analysis. The experiments have been repeated three times. F) Representative bar graph showing relative RNA expression of SPP1, CXCL3, and EREG in THP‐1 cells upon hypoxia treatment by qPCR analysis. The experiments have been repeated three times. G) Representative bar graph showing relative RNA expression of SPP1, CXCL3, and EREG in THP‐1 cells upon HIF1α knockdown by qPCR analysis. The experiments have been repeated three times. H) Bar graph showing SPP1 expression in THP‐1 cells treated with PLIN2 siRNA (siPLIN2) or control (siCtrl) detected by ELISA (n = 3). I) Bar graph showing SPP1 expression in THP‐1 cells treated with HIF1α‐ specific (siHIF1α) or control (siCtrl) siRNA detected by ELISA (n = 3). J) Bar graph showing SPP1 expression in THP‐1 cells harboring PLIN2 overexpression (OE‐PLIN2) or vector (OE‐Ctrl) and transfected with HIF1α‐specific (siHIF1α) or control (siCtrl) siRNA detected by ELISA (n = 3). Data are presented as mean ± SEM. **P < 0.01, ***P < 0.001, ****P < 0.0001 and n.s., not significant. Student's t‐test for B, E, F, one‐way ANOVA analysis for C, G, H–J.

To gain a deeper understanding of the mechanism underlying the promotion of OC metastasis by PLIN2^hi^ macrophages, we delved into the signaling pathways that were differentially regulated in the AAMs. Using the Kyoto Encyclopedia of Genes and Genomes database, we ascertained that HIF1 signaling was the most enriched pathway in AAMs (**Figure** **5**A). This finding prompted us to hypothesize that hypoxia, a hallmark of TME and ascites milieu, might be regulated by PLIN2. Indeed, PLIN2 overexpression induced upregulation of HIF1α in a hypoxic environment treated with 1% O_2_ (Figure 5B; Figure S6M, Supporting Information). Conversely, PLIN2 knockdown reversed HIF1α upregulation induced by hypoxia (Figure 5C; Figure S6N,O, Supporting Information). To confirm that the downstream effector of HIF1α is also influenced by PLIN2, we examined the DEGs in the c4 cluster, among which the alteration of SPP1, EREG, and CXCL3 expression was the most significant (Figure 5D). To validate these findings, we conducted quantitative real‐time PCR analysis, which revealed that the expression of SPP1 mirrored the levels of PLIN2 (Figure 5E–G; Figure S6P, Supporting Information). We further confirmed that SPP1 expression could be effectively repressed by either PLIN2 or HIF1α inhibition (Figure 5H,I; Figure S6Q, Supporting Information). Importantly, HIF1α knockdown abolished the SPP1 upregulation elicited by PLIN2 overexpression (Figure 5J), indicating that HIF1α lies downstream of PLIN2 to induce SPP1 expression.

SPP1 functions as a proverbial secretory protein that exerts pro‐tumor functions.^[^ ^25a^ ^]^ To investigate the role of SPP1 in OC progression and metastasis, we showed that SPP1 knockdown reduced vascular permeability and impeded the migration, invasion, and proliferation of ovarian cancer cells (Figure S7A–F, Supporting Information). These data suggest that SPP1 may function as a downstream regulator of PLIN2 to exert the pro‐tumor effect in OC.

### 2.6 Targeting PLIN2^hi^ AAMs to Harness OC Progression and Ascites Development

**Figure 6:**
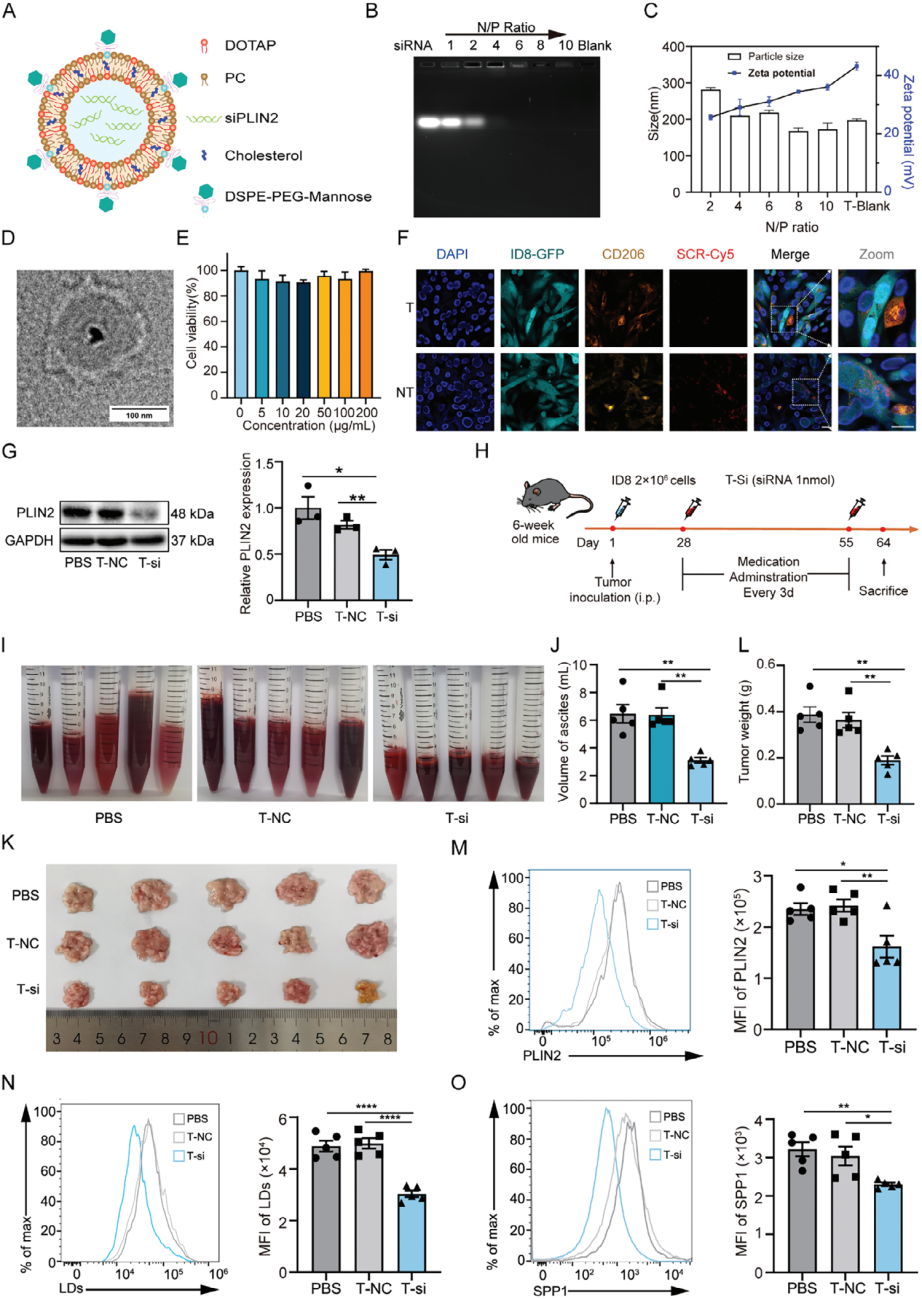
Liposomes incorporating PLIN2‐siRNA attenuate tumor metastasis and ascites formation. A) Scheme depicting the synthesis of the siRNA‐loaded liposome. B) Electrophoretic mobility of siRNA complexed with drug‐free liposomes (T‐blank) in agarose gels by different N/P. C) Effect of different N/P ratios on the particle size and ζ potential of liposomes prepared with siRNA (n = 3). D) Morphology and size of liposomes loaded with PLIN2 siRNA (T‐si; N/P = 6) in PBS revealed by transmission electron microscopy (TEM). E) Cell viability of BMDMs treated with the indicated concentration of liposomes assessed by CCK8 assay (n = 3). F) Representative images of M2‐like macrophages and ID8‐GFP cancer cells co‐incubated with T‐Cy5 (T) or NT‐Cy5 (NT) for 6 h. Red fluorescence, Cy5‐labeled with scrambled siRNA (SCR); green fluorescence, GFP protein expressed in ID8‐GFP cancer cells; orange fluorescence, CD206 staining; and blue fluorescence, nuclei. G) Representative images and quantification of PLIN2 protein expression in cells with indicated treatment assessed by Western blot (n = 3). GAPDH was used as a loading control. T‐si, liposomes loaded with PLIN2 siRNA; T‐NC, liposomes loaded with scramble siRNA. H) Schematic diagram of liposome treatment plan. I) Representative images of ascites obtained from liposome‐treated mice (n = 5). J) Bar graph showing the statistical analysis of (I). K) Tumor metastasis on mesentery were obtained and shown (n = 5). L) Bar graph showing the statistical analysis of (K). M) PLIN2 expression in macrophages of ascites from indicated groups detected by flow cytometry (n = 5). N) Accumulation of LDs in macrophages of ascites from indicated groups detected by flow cytometry (n = 5). O) SPP1 expression in macrophages of ascites from indicated groups detected by flow cytometry (n = 5). Data are presented as mean ± SEM. ^*^ *P* < 0.05, ^**^ *P* < 0.01, ^****^ *P* < 0.0001. One‐way ANOVA analysis for G, J, L–O.

To evaluate the therapeutic potential of PLIN2^hi^ AAMs, we designed liposomes loaded with PLIN2 siRNA that could be delivered to M2 macrophages with mannose expression (T‐si; **Figure** **6**A and Table S6, Supporting Information). As controls, we synthesized M2‐targeting liposomes without siRNA (T‐blank) and M2‐targeting liposomes with scrambled siRNA. SiRNA migration was completely blocked when the N/P ratio was greater than 6, indicating complete siRNA complexation as assessed by agarose gel electrophoresis analysis (Figure 6B). The ζ‐potential of the liposome complex was 23.1 ± 1.6 mV and the average hydrodynamic diameter was 218.5 ± 5.2 nm at an N/P ratio of 6, as determined by dynamic light scattering (Figure 6C). The liposome complex had a uniform spherical morphology in PBS as observed by transmission electron microscopy (TEM) (Figure 6D). Even when incubated with T‐blank at a concentration of up to 200 µg mL^−1^, the survival of BMDMs was not significantly reduced, indicating low cytotoxicity (Figure 6E; Figure S6A, Supporting Information). Since single‐cell transcriptome analysis identified PLIN2^hi^ macrophages with M2‐like features, we sought to verify whether the liposomes could specifically target M2 macrophages. M2‐polarized macrophages and ID8‐GFP cancer cells were mixed and incubated with liposomes loaded with Cy5‐labeled scramble siRNA and coated with mannose (T‐Cy5) or without mannose (NT‐Cy5). Confocal microscopy analysis showed that T‐Cy5 was preferentially taken up by M2 macrophages, whereas NT‐Cy5 exhibited equal levels of uptake in macrophages and cancer cells (Figure 6F). We also observed the uptake of liposomes in various organs in vivo (Figure S6B, Supporting Information).

To validate the efficacy of the liposomal complex, we transfected BMDMs with synthesized T‐si at different time points and showed that both the mRNA and protein levels of PLIN2 were significantly reduced at 72 hours post‐transfection (Figure 6G; Figure S6C, Supporting Information). Importantly, to validate the efficacy of PLIN2‐targeting liposomes in vivo, we constructed a murine OC model by injecting ID8 tumor cells into the peritoneal cavity (Figure 6H). In accordance with the in vitro results, both ascites formation and tumor metastasis were harnessed by PLIN2‐targeting liposomes (Figure 6I–L). Furthermore, such treatment suppressed PLIN2 expression and LD accumulation, which led to the inhibition of downstream SPP1 expression (Figure 6M–O). Collectively, these results underscore the clinical value of targeting PLIN2^hi^ AAMs in the treatment of OC.

## 3 Discussion

Here, we used scRNA‐seq to lay out the immune landscape across multiple sites of HGSOC and discovered a subset of PLIN2^hi^ macrophages that are enriched in ascites and associated with disease progression. PLIN2^hi^ AAMs are abundantly loaded with LDs and can promote OC progression through HIF1α/SPP1 signaling. By targeting PLIN2^hi^ macrophages with liposomes, OC progression and ascites development can be harnessed. This work links lipid metabolism to hypoxia in the context of the ascites immune microenvironment and highlights the active role of ascites in facilitating trans‐coelomic dissemination and cancer progression, providing novel targets for the treatment of OC patients.

Ascites are a unique route of metastasis, as most ovarian cancers spread primarily through the peritoneal cavity. However, the composition and function of the ascites microenvironment is rather complicated and heterogeneous compared to the TME. A study by Izar et al. resolved the immune landscape of 22 ascites samples from 11 HGSOC patients and highlighted the contribution of immune infiltrates and fibroblasts to the subtypes of HGSOC.^[^ ^27^ ^]^ In addition, a recent landmark study by Zheng et al. profiled multiple immune subsets in ascites and deciphered that macrophages in OC ascites were distinct from those in TME by embryonic origin and function.^[^ ^2a^ ^]^ In this study, we identified a novel subset of PLIN2^hi^ macrophages enriched in ascites and confirmed their role in promoting cancer metastasis and ascites production both in vitro and in vivo.

Macrophages in solid tumors have been extensively characterized.^[^ ^5^, ^28^ ^]^ Numerous studies agree that TAMs are associated with tumor progression, metastasis, and chemoresistance. Similarly, macrophages in ascites also facilitate transcoelomic metastasis by participating in spheroid formation.^[^ ^29^ ^]^ A recent work unveiled that macrophages in ascites had different embryonic origins from those in tumors,^[^ ^2a^ ^]^ suggesting the heterogeneity of macrophages in different microenvironment. More interestingly, the ascites microenvironment and cellular ecosystem may differ between pre‐ and post‐menopausal HGSOC.^[^ ^25b^ ^]^ Coincidentally, both of the above studies pointed to SPP1^+^ macrophages as facilitators of immune modulation in ascites. SPP1 was reported to be involved in promoting progression and immune evasion in hepatocellular carcinoma and ovarian cancer.^[^ ^30^ ^]^ In the current study, by comparing single‐cell transcriptomics of immune cells in different lesions of OC, we identified a novel subset of macrophages in the ascitic immune microenvironment. This subset of macrophages exhibited a remarkable resemblance to those found in solid tumors, where they have been observed to promote cancer progression. More importantly, our data showed that PLIN2 might lie upstream of SPP1 to regulate macrophage functions. Further investigations are needed to fully characterize the ontogeny and specific functional profile of these macrophages in relation to their tumor counterparts.

Previous reports revealed that LAMs were enriched in the TME and could fuel cancer progression by promoting proliferation or metastasis.^[^ ^13^, ^31^ ^]^ Recent studies found that PLIN2^hi^ TAMs could sustain tumor growth through CCL6 signaling or suppress T‐cell function through immune checkpoint modulation in prostate cancer and oral squamous cell carcinoma, respectively.^[^ ^10^, ^32^ ^]^ In ovarian cancer, LDs were reported to promote cancer cell stemness via stearoyl‐CoA desaturase 1.^[^ ^33^ ^]^ However, the regulatory mechanisms of LDs in LAMs remain to be elucidated. A body of research has indicated that hypoxia signaling can modify lipid metabolism through a HIF‐dependent mechanism.^[^ ^34^ ^]^ Nevertheless, we demonstrated that LDs were accumulated in PLIN2^hi^ LAMs and that PLIN2 in macrophages could positively regulate HIF1α, thereby exacerbating hypoxia in ascites. The findings of this study indicate the presence of a reverse regulatory axis, whereby the accumulation of LDs in macrophages serves as a determining factor in the regulation of hypoxic signaling. These results contribute to a more intricate understanding of the regulatory mechanisms that govern the hypoxic microenvironment in cancer settings and provide a novel perspective on the relationship between lipid metabolism and hypoxia.

In conclusion, our comprehensive characterization of the immune microenvironment in multiple sites of OC reveals a subset of PLIN2^hi^ macrophages that are enriched in ascites and promote ascitic development and cancer metastasis through HIF1α/SPP1 signaling. Our findings establish a link between LAMs and hypoxia in ascites and provide novel therapeutic targets for the treatment of malignant ascites.

## 4 Experimental Section

### Human Specimen

Seven patients diagnosed with HGSOC and six patients diagnosed with benign gynecological tumors were enrolled in this study. Nineteen samples were collected from ten patients, including fresh tumors, metastatic tumors, ascites, and corresponding non‐malignant tissue, and profiled by scRNA‐seq. Three patients had matched tissues from multiple sites. The clinical information of all patients is summarized in Table S1 (Supporting Information). All samples from humans were obtained via a dedicated protocol approved by the Medical Ethics Committee of the Fifth Affiliated Hospital of Sun Yat‐sen University (2022‐K319‐1) and the Medical Ethics Committee of the Sun Yat‐Sen University Cancer Center (202211082310000127180). All patients in this study provided written informed consent for sample collection and data analysis.

### Animal Model Construction and Treatment

Animals were maintained under pathogen‐free conditions in the Guangdong Provincial Engineering Research Center of Molecular Imaging in accordance with the guidelines of the Animal Ethics Committee of the Fifth Affiliated Hospital of Sun Yat‐sen University (Approval numbers: 00248, 00345, 00457, and 00487). Six‐week‐old female C57BL/6 mice were injected intraperitoneally with 1.5 × 10^6^ ID8 cells. Eight weeks after tumor inoculation, the animals were euthanized and ascites were collected. 2.5 mL of PBS was used to flush the peritoneal cavity of control animals. Centrifugation was performed to obtain cells and supernatant, followed by a flow cytometry assay. For the intervention experiment, after 28 days of tumor inoculation, the liposomal drug containing siRNA targeting PLIN2 or scramble siRNA (1 nmol) or PBS solution was injected into the peritoneal cavity, and thereafter the drug was administered every three days until the mice were sacrificed on day 64 or humane endpoints such as reduction of body weight (>20%) or loss of appetite (>50%).

For macrophage reinfusion assay, 1.5 × 10^6^ ID8 cells were peritoneally injected with differential expression of PLIN2 by means of stable knockdown of PLIN2 in RAW264.7 or overexpression of PLIN2 in J774A.1. Four (for overexpression experiment) or six weeks (for knockdown experiment) after tumor inoculation, mice were sacrificed and ascites and tumors were collected and photographed.

The B6C3F1 mouse (6–8 weeks old, female) was generated by crossing a male C3H mouse and a female C57BL/6 mouse. Mice were randomly divided into two groups. RAW264.7 macrophages with stable PLIN2 knocked down or control shRNA mixed with HM1 cells (1×10^6^ cells respectively in 100 µL PBS) were injected into the peritoneal cavity. Mice were weighed and observed every other day and sacrificed four weeks post‐inoculation for the collection of ascites and tumors. Data were analyzed under double‐blind conditions.

### Cell Lines and Cell Culture

Human umbilical vein endothelial cells (HUVECs) were obtained from SciencCell (USA). The murine endothelial cell line C166 and the murine macrophage cell line RAW264.7 were obtained from ATCC. Mouse ovarian epithelial cancer cells (ID8) were purchased from zqxzbio (China). Bone marrow‐derived macrophages (BMDMs) were extracted from C57BL/6 mouse bone marrow. The human monocyte cell line THP‐1 was a gift from Professor Dong‐Ming Kuang (Sun Yat‐sen University). SK‐OV‐3 cell line was a gift from Professor Min Feng (Sun Yat‐sen University). U937 and OVCAR‐3 cell lines were purchased from (Procell, China).

HUVECs were cultured in ECM (SciencCell, USA) supplemented with 5% FBS, 1% EC growth supplement (ECGS), 100 U mL^−1^ penicillin, and 100 µg mL^−1^ streptomycin. OVCAR‐3 cells were cultured in RPMI 1640 medium (Gibco, USA) supplemented with 20% FBS (Procell, China), 100 U mL^−1^ penicillin, and 100 µg mL^−1^ streptomycin (Beyotime, China), 10 µg mL^−1^ Insulin (Procell, China). SK‐OV‐3 was cultured in McCoy's 5A (Procell, China) with 20% FBS (Procell, China), 100 U mL^−1^ penicillin, and 100 µg mL^−1^ streptomycin (Beyotime, China). THP‐1 and U937 cells were cultured in RPMI 1640 medium (Gibco, USA) supplemented with 10% FBS (Procell, China), 100 U mL^−1^ penicillin, and 100 µg mL^−1^ streptomycin (Beyotime, China). 0.1 µg mL^−1^ PMA (Solarbio, China) was used to stimulate THP‐1 and U937 cells into macrophages for 24 h followed by indicated treatment such as transfection. C166, BMDM, and RAW264.7 cells were cultured in DMEM (Gibco, USA) supplemented with 10% FBS, 100 U mL^−1^ penicillin, and 100 µg mL^−1^ streptomycin. All cells were maintained in a humidified incubator with 5% CO_2_ that was at 37 °C.

### Extraction and Transfection of Bone Marrow‐Derived Macrophages

6–8‐week‐old C57BL/6 mice were sacrificed and placed on a sterilized operating table, and the hind legs were pulled to dislocate the femur from the hip bone. Then, the leg muscles were removed to expose the femur and tibia. After removing the bones below the knee joint by cutting the ligaments, the separated femurs and tibias were washed in pre‐cooled saline. Both ends of the femur and tibia were cut, and the marrow cavities were rinsed three times with 1640 medium containing 10% FBS. The cells were collected and treated with red blood cell lysis buffer for 2 min, then cultured with 10 mL of DMEM medium containing 10% FBS, 1% penicillin‐streptomycin, 10 ng mL^−1^ M‐CSF (NovoProtein, China). After 7 days, 10 ng mL^−1^ IL‐4 (PeproTech, USA) was added for two days to polarize cells into M2‐like macrophages.

SiRNA was transfected into cells using Lipofectamine RNAiMAX (Thermofisher, USA)‐mediated delivery according to the manufacturer's instructions. All siRNA sequences were summarized in Table S6 (Supporting Information). Briefly, cells were extracted and plated into a 6‐well plate. siRNA‐RNAiMAX complexes were prepared and added to the cells for 8 h, followed by culturing in full medium for 48 h, followed by the indicated assays.

### Cell Migration and Invasion Assays

BMDM, U937 or THP‐1 cells with PLIN2 knockdown or overexpression were seeded into the lower inserts of transwell plates (Corning, USA), and ID8, OVCAR‐3 or SK‐OV‐3 cells (1 × 10^4^) were seeded in the transwell chambers with the pore size of 8 µm (Corning, USA), and the cells were cultured for 24 h. Cells that migrated to the lower surface were stained with crystal violet (Beyotime, China) and quantified by counting 3 randomly selected microscopic fields at × 200 magnification. The same method was applied to the invasion assay except that the upper chambers were coated with 250 µg mL^−1^ matrix gel (ABW, China).

### Permeability Assay

1 × 10^5^ HUVECs or C166 cells were seeded on the top of inserts in a 24‐well transwell chamber with a pore size of 0.4 µm and cultured for 24 h until mono‐layer of cells was achieved. 5 × 10^4^ THP‐1, U937 or BMDM cells from different groups were added to the endothelial layer. After co‐culturing for 24 h, the chamber was washed twice with 100 µL PBS. Then, 500 µL PBS was added to the lower layer and 200 µL 2 mg mL^−1^ TRITC‐dextran‐70 kDa (Sigma, USA) to the upper layer. The entire transwell was incubated at 37 °C for 2 h. The liquid in the lower compartment was collected and analyzed using a fluorescence spectrophotometer (PerkinElmer, Envision, USA).

For the in vivo permeability assay, 1.5 × 10^6^ ID8 cells with differential expression of PLIN2 by stable knockdown of PLIN2 in RAW264.7 were injected peritoneally. Two weeks after tumor inoculation, mice were assessed for peritoneal vascular permeability. 100 µL 10 mg mL^−1^ TRITC‐dextran‐70 kDa (Sigma, USA) was injected intravenously. 30 min later, the mice were sacrificed and injected intraperitoneally with 2.5 mL PBS. The fluid in the peritoneal cavity was collected and analyzed using a fluorescence spectrophotometer (PerkinElmer, Envision, USA).

### Electrical Resistance Analysis

The RTCA‐DP xCELLigence system (Roche Applied Science ACEA Biosciences, USA) was used. In this case, HUVEC cells were seeded on E‐16 plates at a density of 1 × 10^4^ cells per well in an appropriate culture medium. After 24 h, 1 × 10^4^ macrophages per well were seeded on E‐16 plates and incubated with HUVECs for 24 h to observe changes in cell index (CI). Normalized cell index (nCI) was calculated by dividing the normalized time CI by the original CI value.

### Flow Cytometry

Cells from the mouse peritoneal cavity or patient ascites were filtered through a 70 µm filter. Red blood cells in the cell suspension were lysed using red blood cell lysis buffer, and the supernatant was removed by centrifugation. Then, cells were washed with PBS and stained for flow cytometric analysis with the following antibodies and dyes: Nile Red (Abmole, USA), BODIPY 493/503 (GLPBIO, USA), LIVE DEAD FIX 808/876 (Thermofisher, USA), h‐Plin2‐AF647 (Abcam, USA), h‐CD45‐BV605 (BioLegend, USA), h‐CD11b‐PE (BioLegend, USA), h‐CD68‐BV421 (BioLegend, USA), m‐Plin2‐AF647 (NOVUS, USA), Zombie UV Fixable Viability Kit (BioLegend, USA), m‐CD45‐BV780 (Thermofisher, USA), m‐F4/80‐PE (BioLegend, USA). To obtain macrophages from ascites, human ascites were centrifuged, followed by red blood cell lysis. CD45‐FITC (BD, USA) and CD14‐PE (BioLegend, USA) were used to stain the cells, and CD14^+^ macrophages were sorted by BD FACSAria Fusion (BD Biosciences, USA).

### Enzyme‐Linked Immunosorbent Assay (ELISA)

SPP1 secreted by cells was measured by the mouse SPP1 ELISA kit (RayBio, USA). Briefly, 1 × 10^6^ macrophages were cultured in a 6‐well round‐bottom plate in a 2 mL medium for 24 h. The concentration of SPP1 in the supernatants was determined according to the manufacturer's protocol. Briefly, the standard curve was plotted according to the instructions. Samples were prepared and the absorbance OD450 of each sample was measured. Subsequently, the concentrations were calculated according to the standard curve.

### Western Blot

Total cell lysates were extracted from cells and the protein concentration was detected by BCA assay kit (Beyotime, China). Total cell lysates were resolved on 10% SDS‐PAGE and transferred to PVDF membrane (Merck Millipore, Germany). The membrane was then incubated with primary antibody overnight at 4 °C, followed by being incubated with HRP‐conjugated secondary antibody (abcam, USA) for 1 h at room temperature. Bands were visualized by ECL and imaged with ChemiDoc XRS (Bio‐Rad, USA). β‐actin or GAPDH was used as an internal control. All primary antibodies used in this study are summarized in Table S7 (Supporting Information).

### Transfection and Establishment of Stable Cell Lines

PLIN2 stable knockdown in RAW264.7 cells or stable overexpression in J744.1A cells were established by infecting cells with a lentiviral vector (Genechem, China). Cells were infected with lentivirus at MOI 25 using 0.8 µg mL^−1^ polybrene. Stable cell lines were obtained by selection with 5 µg mL^−1^ puromycin (Solarbio, China) selection for 2 weeks, and the transfection efficiency was observed by an inverted fluorescence microscope. The efficiency of RNA interference was assessed via qRT‐PCR and Western blot. The same way was used to accomplish the PLIN2 stable overexpression of THP‐1 cells.

### RNA Isolation and Quantitative Real‐Time PCR

Total RNA was extracted using Total RNA Kit I (Omega, USA). First‐strand cDNA was synthesized from 2 µg total RNA using HiScript III RT SuperMix (Vazyme, China). Amplification and detection were performed using the ABI QuantStudio 7 Flex (Thermofisher, USA). Quantitative PCR conditions were as follows: 95 °C for 10 s, followed by 40 cycles of 95 °C for 10 s and 60 °C for 10 s, and finally 95 °C for 15 s, 60 °C for 30 s and 95 °C for 15 s. The comparative cycle threshold (Ct) method was used to analyze the relative expression of specific mRNAs. Primer sequences are listed in Table S8 (Supporting Information).

### Immunofluorescence and Confocal Analysis

THP‐1 and BMDM cells were treated with ascites or as indicated. The cells were then fixed with 4% formaldehyde in PBS for 5 min at room temperature and blocked with 1% BSA for 30 min, followed by incubation with BODIPY (493/503) or Nile Red solution for 10–15 min. Afterward, the samples were counterstained with a fluorescence‐anti‐quenching solution containing DAPI (Solarbio, China) for 10 min and observed with a confocal microscope (Zeiss, Germany). In a co‐culture study, macrophages and cancer cells expressing green fluorescent protein (ID8‐GFP) were co‐incubated with T‐Cy5 or NT‐Cy5 in the DMEM medium. After incubation for 6 h, the cells were treated with 4% paraformaldehyde for 15 min. The M2‐like macrophages were stained with fluorescence‐labeled anti‐CD206 antibody (R&D, USA), and the nuclei were stained with DAPI. The siRNA uptake and distribution in two different cells were observed under a confocal microscope to evaluate the binding specificity of liposomes.

### Preparation of Liposome

DOTAP (Macklin, China), PC (Aladdin, China), CHOL (SEIKO, Japan), and DSPE‐PEG‐Mannose/DSPE‐PEG (Tanshtech, China), were dissolved in 10 mL of CHCl_3_ followed by spin evaporation at 40 °C to form a thin film. DEPC water was added and hydrated by ultrasonication at 350 W for 10 min. The solution was filtered through a syringe filter (pore size: 450 nm) to remove large aggregates and to obtain the liposome solution (T‐blank). The non‐targeting blank liposome (NT‐blank) was prepared similarly without adding DSPE‐PEG. According to the pre‐designed N/P ratio, a certain amount of PLIN2 siRNA or scramble siRNA (RiboBio, China) was added to the T‐blank and NT‐blank solution, and then the solution was incubated at room temperature for 30 min to obtain the siPLIN2‐ or siSCR‐ loaded liposome (T‐si & NT‐si, T‐SCR & NT‐SCR). Otherwise, Cy5‐labeled SCR was used at an N/P ratio of 6 to form the liposome of T‐Cy5 and NT‐Cy5 for cell uptake study.

### Characterization of Liposome Drugs

The size distribution and ζ potential of micelleplexes at 25 °C were analyzed by dynamic light scattering using the Malvern Zetasizer Nano ZS equipment with a detection angle of scattered light at 90° and 15°, respectively. Each sample was measured in triplicate. Scrambled siRNA (SCR) was centrifuged at a rate of 12000 g for 2 min and dissolved in sterilized water. 0.5 µg of SCR siRNA was mixed with T‐blank at different N/P ratios and incubated at room temperature for 30 min. The mixture was loaded onto a 1% agarose gel at 0.5 µg mL^−1^ with Ethidium bromide and underwent electrophoresis in TAE (Tris‐acetate‐EDTA) buffer at a voltage of 80 V for 30 min. The retardation of siRNA mobility was observed and recorded using a gel imaging system.

### Cytotoxicity Assay

The M2‐like macrophages were cultured in a DMEM medium containing 10% FBS, 1% penicillin‐streptomycin, 10 ng mL^−1^ MCS‐F, and 10 ng mL^−1^ IL‐4. M2‐like macrophages (1 ×10^4^) were incubated in a 96‐well plate. Then, Cell Counting Kit‐8 (APExBIO, USA) solution was added to each well and incubated for 2 h to evaluate cell proliferation. The absorbance of each well was measured at OD 450 using a fluorescence spectrophotometer from day 1–3. CCK8 kit was used to detect ID8 proliferation treated with the supernatants from RAW264.7 after SPP1 knockdown.

### Statistical Analysis

Data are shown as mean ± SEM. Statistical analyses are performed as indicated in the figure legends using GraphPad Prism 9.5. Differences are considered significant at *P* < 0.05. Graph of TOC (Table of Contents) were created with biorender.com.

### Data and Materials Availability

All materials used in the study are commercially available according to the product number or request to the corresponding authors for a probable reason. The scRNA‐seq data and bulk‐seq data reported in this paper had been deposited in the Genome Sequence Archive (https://ngdc.cncb.ac.cn/gsa‐human) and are available under the accession number HRA002362.

## Conflict of Interest

The authors declare no conflict of interest.

## Author Contributions

H.L., X.S., Y.Z., and B.X. contributed equally to this work. H.H., H.L., X.S., Y.Z., H.N., X.C., J.L. did conceptualization. H.L., X.S., Y.Z., S.Z., Q.L., Y.Z., S.G. did methodology. H.L., X.S., Y.Z., B.X., Y.J., J.H., S.H., P.D. did investigation. H.L., X.S., Y.Z., B.X., S.Z., Y.J., Y.D., J.H., L.W., Q.Z., Y.Z., P.D. did visualization. H.H., S.G., Z.Y. did funding acquisition. H.H., F.W., J.L., H.N. did project administration. H.H., H.N., X.C., J.L. did supervision. H.L., Y.Z., X.S., Q.L. wrote the original draft. H.L., Y.Z., H.H., H.N., X.C., J.L. reviewed and did editing.

## Supporting information



Supporting Information

Supplemental Table 1

Supplemental Table 2

Supplemental Table 3

Supplemental Table 4

Supplemental Table 5

Supplemental Table 6

Supplemental Table 7

Supplemental Table 8

## Data Availability

The data that support the findings of this study are openly available in Genome Sequence Archive at https://www.ngdc.cncb.ac.cn/gsa‐human, reference number 2362.
