# Supplementary material for: PLIN2 Promotes Lipid Accumulation in Ascites‐Associated Macrophages and Ovarian Cancer Progression by HIF1α/SPP1 Signaling
Source: Adv Sci (Weinh). 2025 Feb 7;12(12):2411314. doi: 10.1002/advs.202411314 (PMC11948008; doi:10.1002/advs.202411314)
Supplement: Supplementary file 1 — Supporting Information [file ADVS-12-2411314-s003.docx]

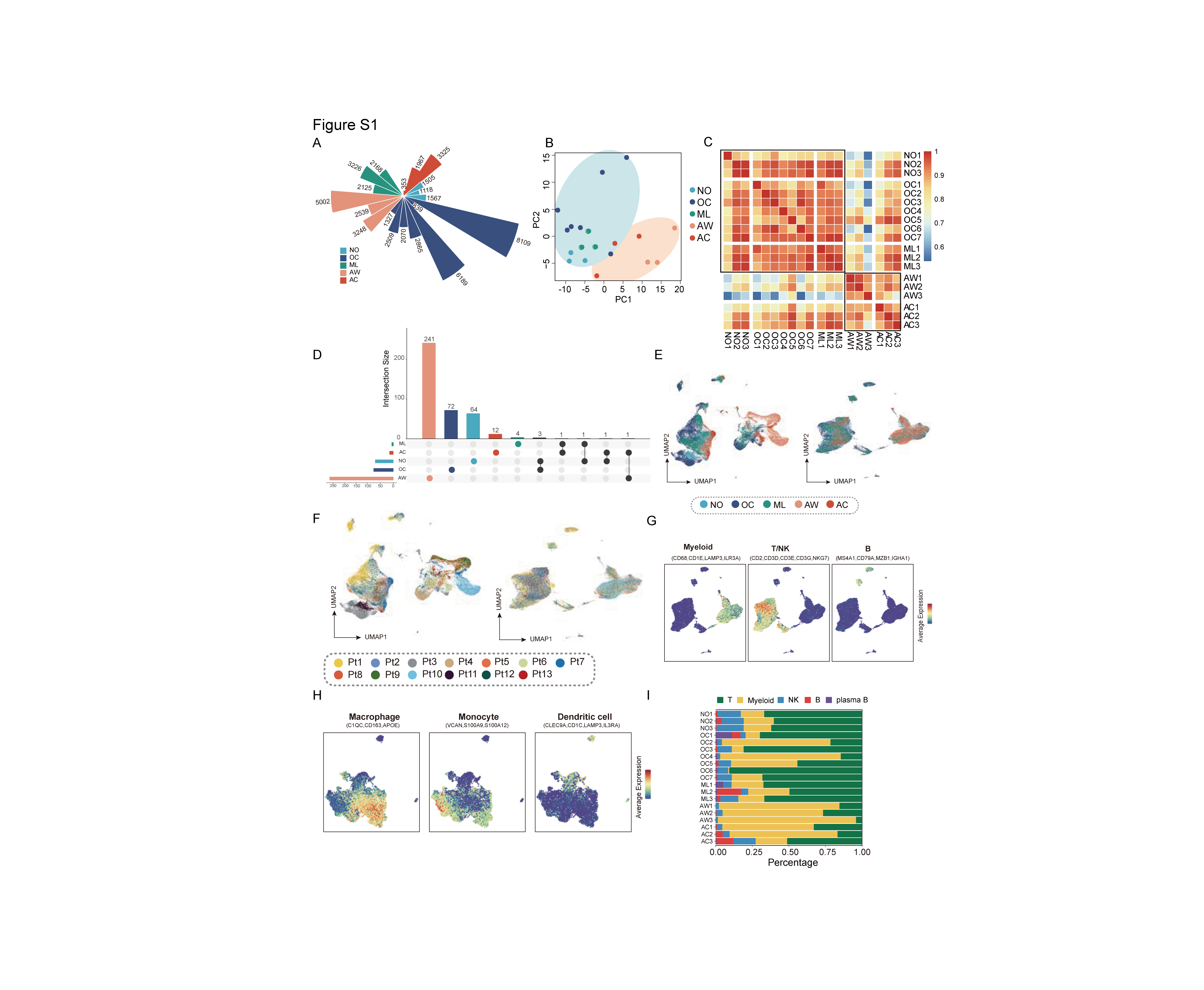


**Figure S1. Immune landscape of multiple lesions in HGSOC.**

**A)** Nightingale Rose Charts showing the cell number of different lesions. **B)** PCA analysis displaying the transcriptomic difference of multiple lesions. NO, normal ovary; OC, ovarian cancer primary tumor; ML, metastatic lesion; AW, abdominal washing; AC, ascites. The different color ellipse indicates the tissue with similar transcriptome. **C)** Heatmap with the correlation analysis among 19 samples. **D)** Upset plot clarifying the unique high expression gene number among five tissues. **E-F)** Seurat analysis of combined CD45^+^ immune cells from 19 samples with each color coded to tissues (E) and patient ID (F). **G)** UMAP plot panels showing the curated gene set expression of immune cell types. **H)** UMAP plot panels showing the curated gene set expression of myeloid cell types. **I)** Stacked bar chart specifying the percentage of cell types in all 19 lesions.


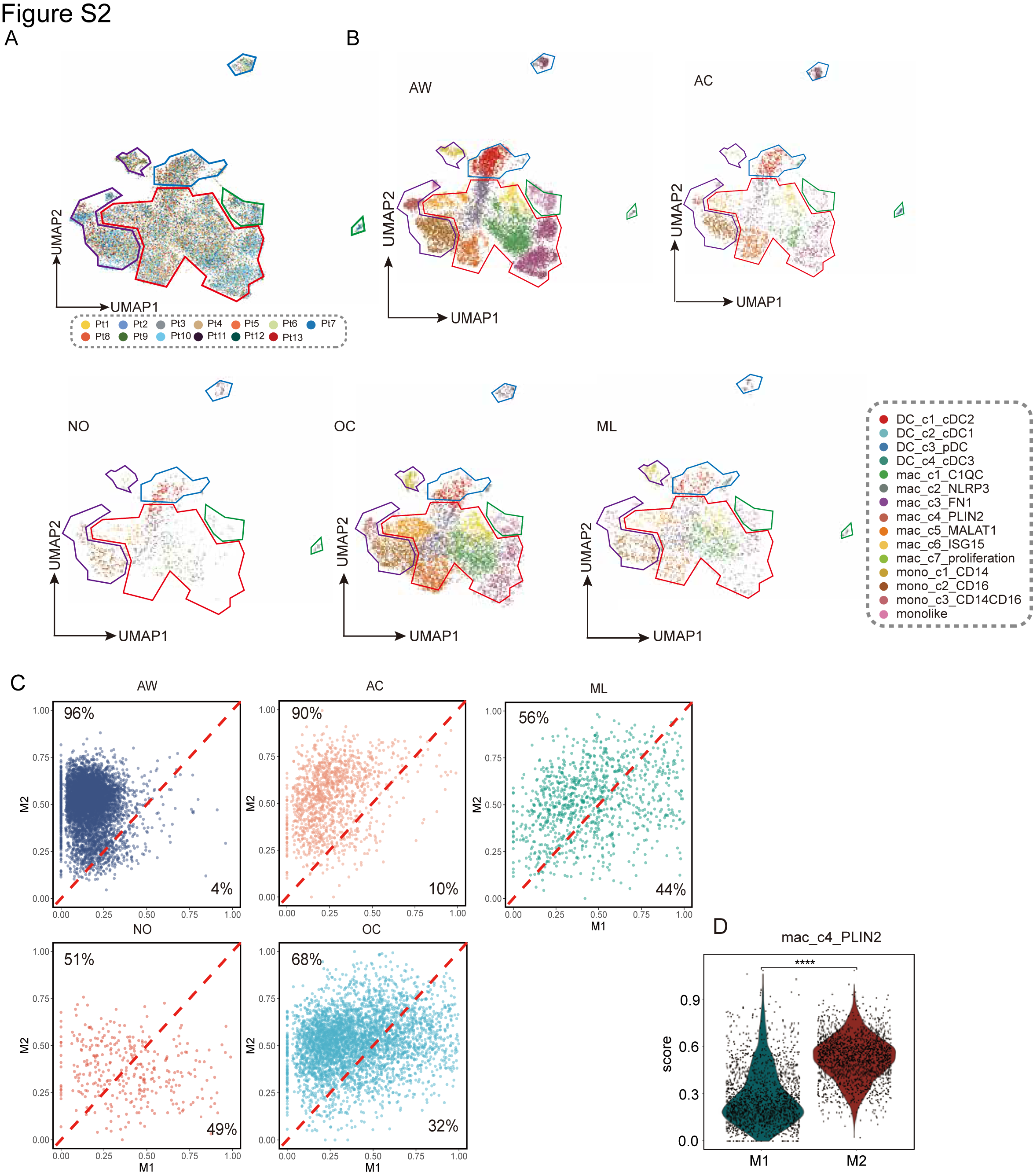


**Figure S2. The tissue preference of myeloid cell clusters and polarization status of macrophages.**

**A)** UMAP visualization of myeloid cells labeled by patient ID. **B)** UMAP plot showing subsets of myeloid cells in different tissues with color coded by clusters. **C)** The macrophage activity and polarization evaluated by M1 and M2 signatures in different sites. **D)** Violin plots displaying the expression of genes related to classical M1 and M2 macrophages using unpaired Student’s t test, **** indicates p<0.0001.


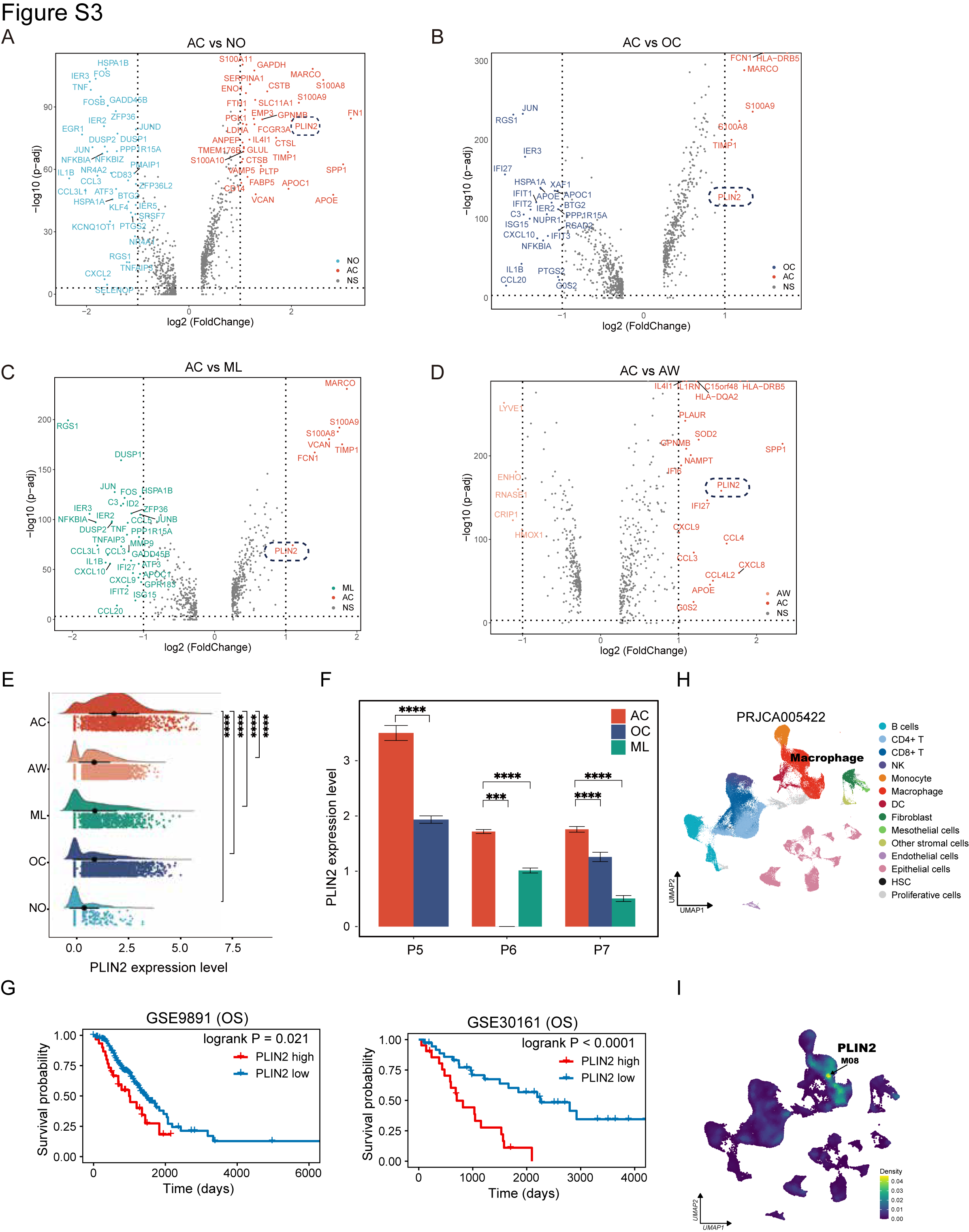


**Figure S3. Expression of PLIN2 in macrophages in different tissues.**

**A-D)** Differentially expressed genes of macrophages in ascites compared with normal ovary, primary tumor, metastatic lesion and normal abdominal washing. Dashed line marks the significant threshold (logFC > 1; -log10 (adjusted p value) > 0.05). **E)** Violin plot depicting the expression level of PLIN2 in macrophages from different lesions. **F)** Grouped bar plot specifying the PLIN2 expression in macrophages from different lesions in the same patient. **G)** Kaplan-Meier plots illustrating overall survival (OS) in patients with ovarian cancer in GSE9891 and GSE30161 cohort stratified by PLIN2 expression. The cutoff point was determined by maximizing the log-rank test p value between survival curves. **H)** UMAP plot exhibiting the different cell types of PRJCA005422 cohort. **I)** UMAP plot exhibiting the expression of PLIN2 in all cell types. One-way ANOVA for E, unpaired Student’s t test for F and log-rank test for G.


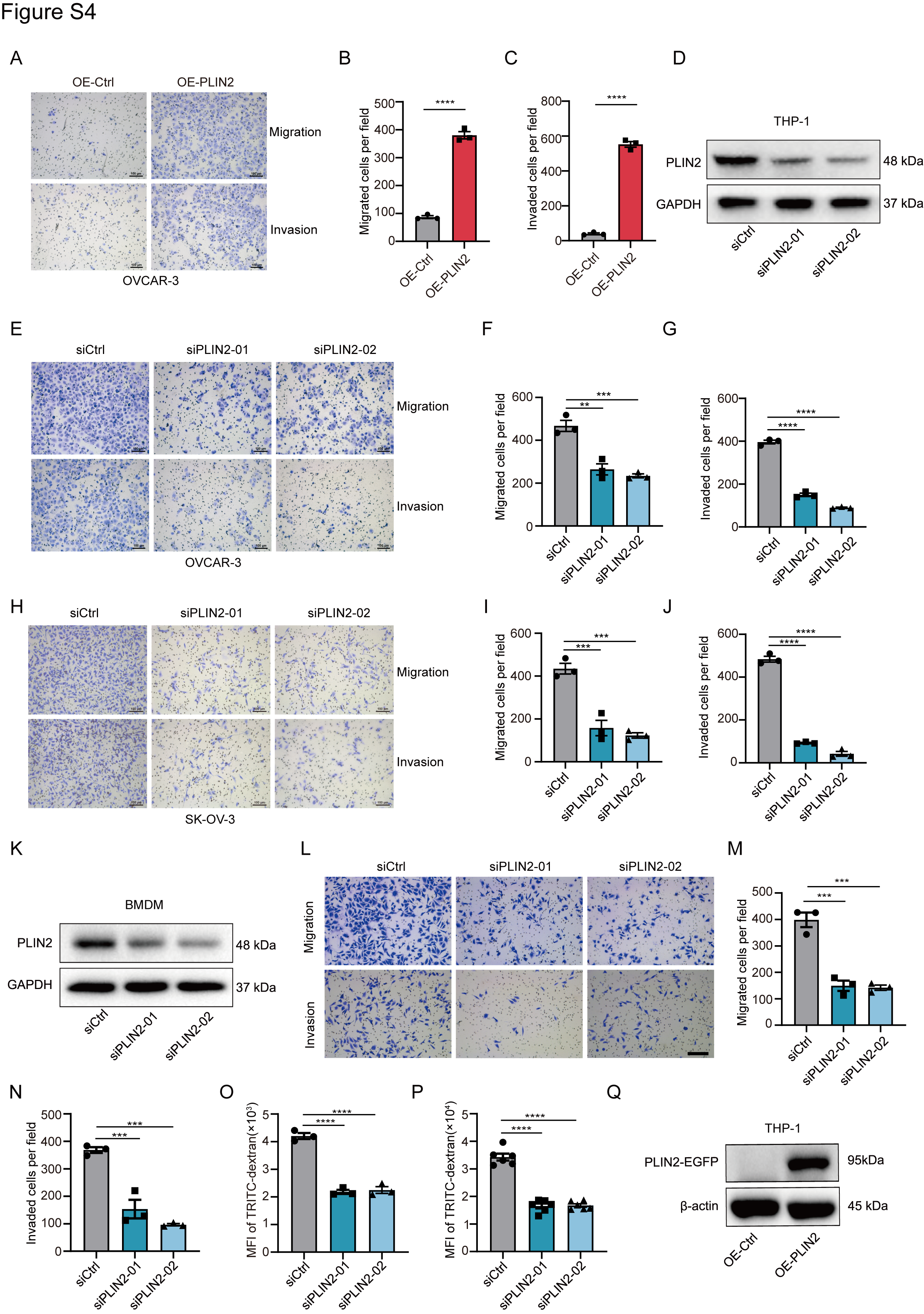
**Figure S4. PLIN2^hi^ macrophages promote vascular permeability and tumor metastasis.**

**A)** Representative graph of migration and invasion assay of OVCAR-3 indirectly cocultured with THP-1 macrophages with overexpression of PLIN2 or not (n=3). The scale bars represent 100 μm. **B-C)** Bar graph showing the statistical analysis of (A). **D)** Knockdown efficiency of siRNA targeting PLIN2 in THP-1 was validated by Western blot assays. GAPDH was used as a loading control (n=3). **E)** Representative graph of migration and invasion assay of OVCAR-3 indirectly cocultured with U937 macrophages that were transiently transfected with PLIN2-specific siRNA or control siRNA (n=3). The scale bars represent 100 μm. **F-G)** Bar graph showing the statistical analysis of E. **H)** Representative graph of migration and invasion assay of SK-OV-3 indirectly cocultured with U937 macrophages that were transiently transfected with PLIN2-specific siRNA or control siRNA (n=3). The scale bars represent 100 μm. **I-J)** Bar graph showing the statistical analysis of (H). **K)** Knockdown efficiency of siRNA targeting PLIN2 in BMDM was validated by Western blot assays. GAPDH was used as a loading control (n=3). **L)** Representative graph of migration and invasion of ID8 indirectly cocultured with BMDM cells that were transiently transfected with PLIN2-specific siRNA or control siRNA (n=3). The scale bars represent 200 μm. **M-N)** Bar graph showing the statistical analysis of (L). **O)** TRITC-dextran tracer fluorescence from U937 and endothelial cells (HUVEC) coculture systems in which macrophages transiently transfected with PLIN2-specific siRNA or control siRNA (n=3). **P)** TRITC-dextran tracer fluorescence from BMDM and endothelial cells (C166) coculture systems in which macrophages transiently transfected with PLIN2-specific siRNA or control siRNA (n=6). **Q)** The overexpression of PLIN2 in THP-1 was validated by western blot assays. GAPDH was used as a loading control (n=3). Data are mean ± SEM. **P < 0.01, ***P < 0.001 and ****P < 0.0001. One-way ANOVA analysis for G-J and M-P. Unpaired Student’s t test for B and C.


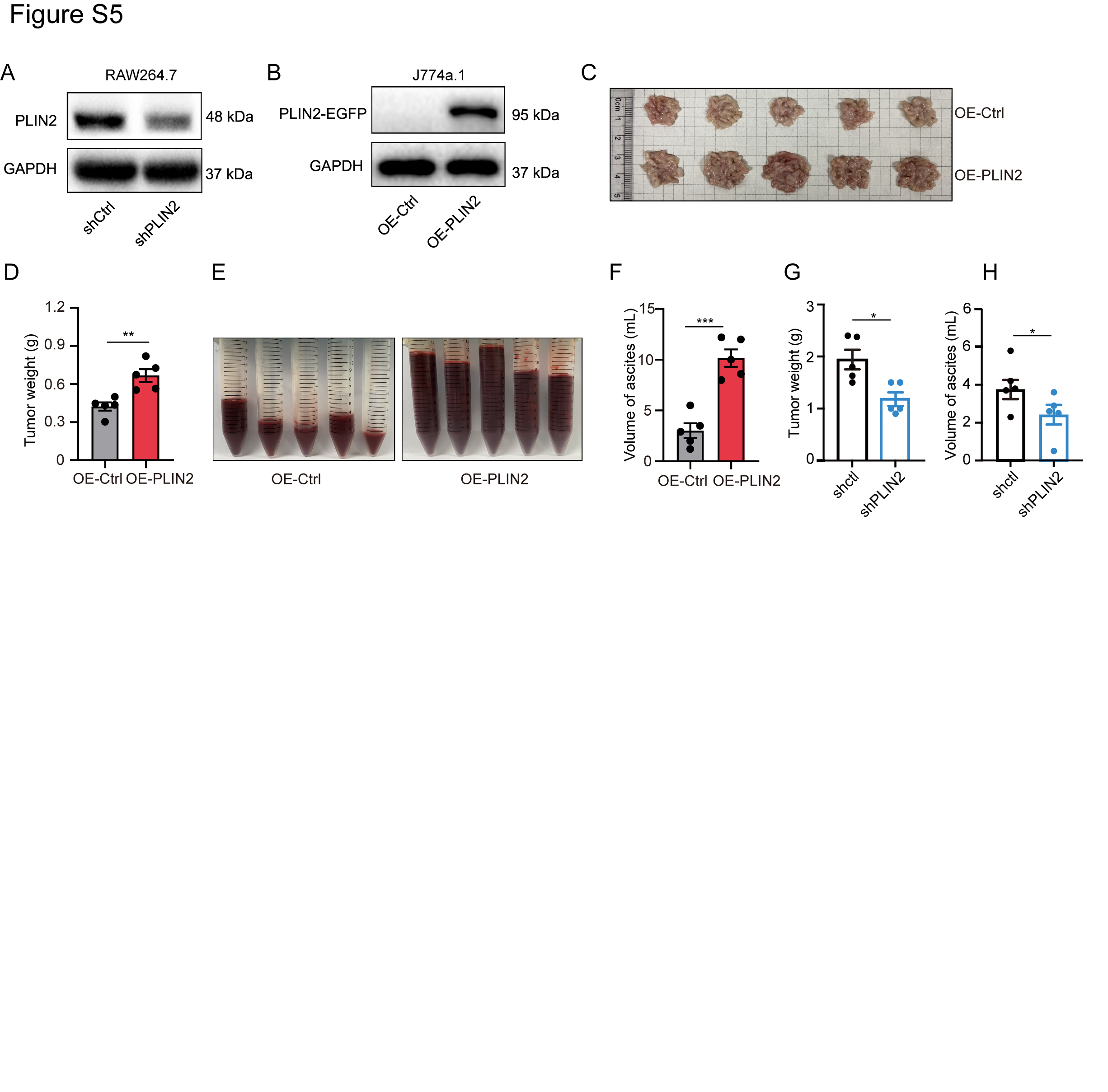


**Figure S5. PLIN2^hi^ macrophages promote ascites development in OC metastasis mouse models.**

**A)** Knockdown efficiency of sRNA targeting PLIN2 in RAW264.7 was validated by Western blot assays. GAPDH was used as a loading control. **B)** The overexpression of PLIN2 in J744A.1 was validated by Western blot assays. GAPDH was used as a loading control (n=3). **C)** Intraperitoneal injection of ID8 and RAW264.7 (with or without PLIN2 overexpression) into mice (n=5 per group), and tumor metastasis on mesentery were obtained. **D)** Bar graph showing the statistical analysis of (C). **E-F)** Volume of ascites were photographed (E) and statistical analysis (F) were conducted (n=5). **G)** Intraperitoneal injection of HM1 and RAW264.7 (with or without PLIN2 knockdown) into mice (n=5 per group), and tumor weight from each group was statistically analyzed. **H)** Statistical analysis of ascites volume obtained from model of G. Data are mean ± SEM. *P < 0.05 **P < 0.01; ***P < 0.001; ****P < 0.0001. Unpaired Student’s t test D, F-H.


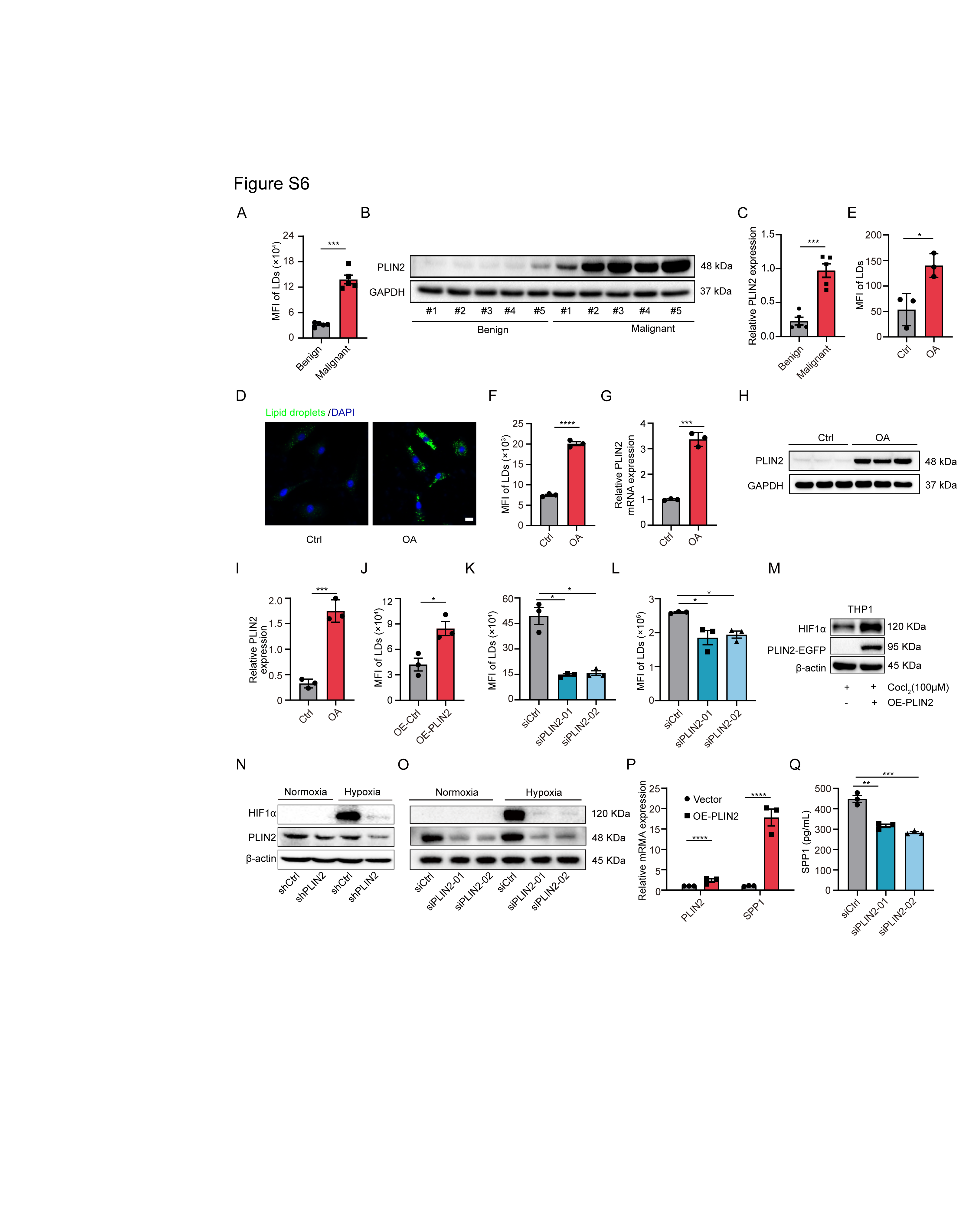


**Figure S6. PLIN2 regulates LDs and HIF1α/SPP1 signaling in macrophages.**

**A)** Bar graph showing statistical analysis of LDs accumulation in THP-1 macrophages after incubation with ascites from benign or malignant patients detected by flow cytometry (n=5). **B)** PLIN2 expression in BMDM after incubation with ascites from benign or malignant patients detected by Western blot analysis. GAPDH was used as a loading control (n=5). **C)** Statistical analysis of (B). **D, E)** Representative picture and statistical analysis of LDs accumulation (green signals indicate LDs, blue signals indicate nuclei in BMDM with or without OA treatment by confocal microscopy (n=3), the scale bars represent 10 μm. **F)** Flow cytometry analysis of PLIN2 level in BMDM with or without OA treatment by qPCR (n=3). **H, I)** Western blot and statistical analysis of PLIN2 expression in BMDM cells after OA treatment. GAPDH was used as a loading control (n=3). **J, K)** Bar graph showing statistical analysis of LDs accumulation in THP-1 macrophages which were overexpressed (J) or knocked down (K) with PLIN2 detected by flow cytometry (n=3). **L)** Bar graph showing statistical analysis of LDs accumulation in BMDM macrophages which were knocked down PLIN2 detected by flow cytometry (n=3). **M)** Examination of HIF1α expression in the context of PLIN2 overexpression under the environment of CoCl_2_ (100 μM) in THP-1 cells by Western blot. β-actin was used as a loading control (n=3). **N)** Examination of HIF1α expression in context of PLIN2 knockdown under normoxia and hypoxia (1% O_2_) environment in RAW264.7 cells by Western blot. β-actin was used as a loading control (n=3). **O**) Representative images and statistical analysis of HIF1α expression upon PLIN2 knockdown under normoxia and hypoxia (1% O_2_) environment in THP-1 cells by Western blot. β-actin was used as a loading control (n=3). **P, Q**) Validation of SPP1 expression in the context of PLIN2 overexpression in RAW264.7 cells (P) or knockdown in BMDM cells (Q) by qPCR and ELISA (n=3). Data are mean ± SEM. *P < 0.05 **P < 0.01; ***P < 0.001; ****P < 0.0001. Unpaired Student’s t test for A, C, E-G, I, J, P. One-way ANOVA analysis for K, L, Q.


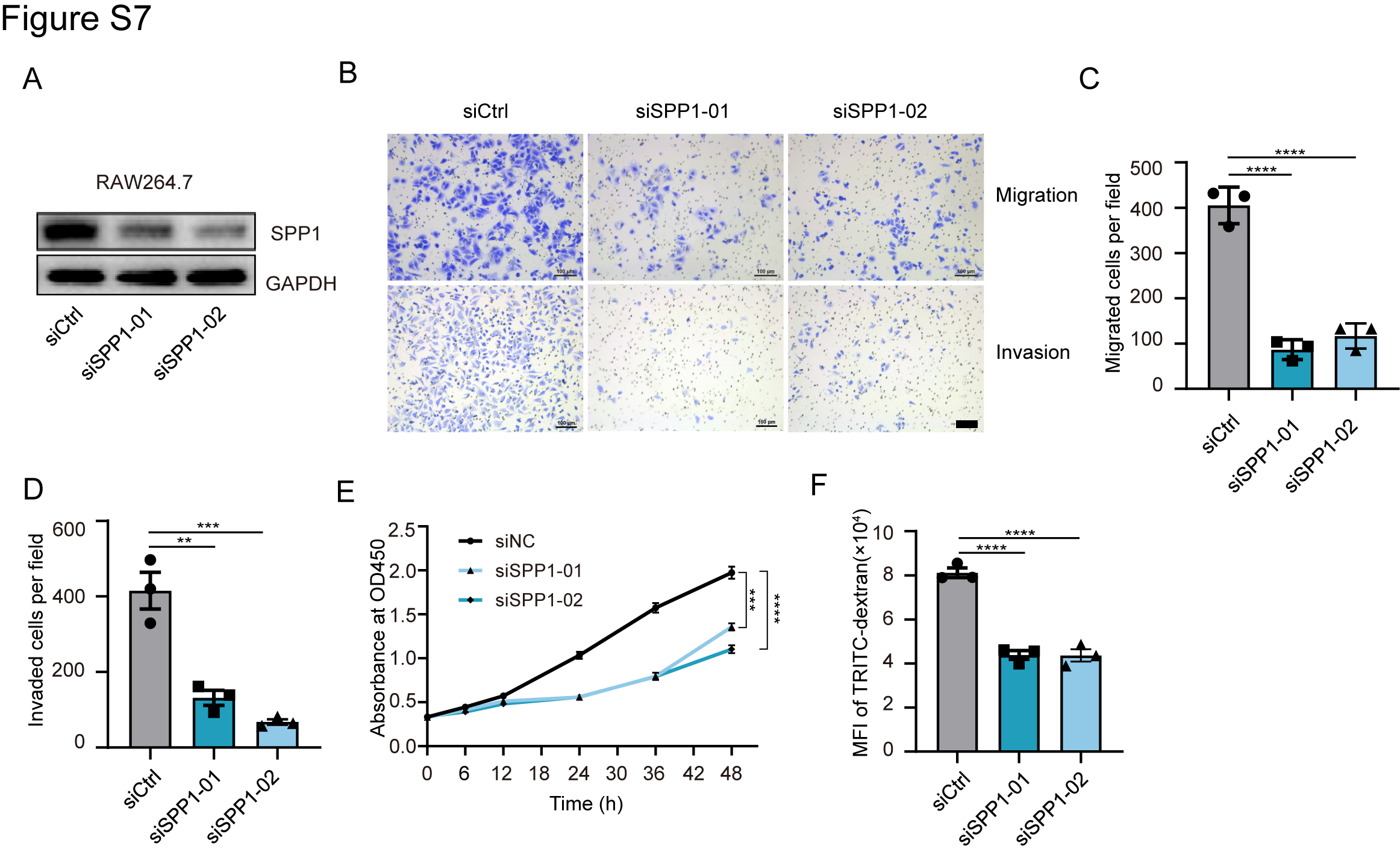


**Figure S7. The effect of SPP1 on tumor metastasis and vascular permeability.**

**A**) Knockdown efficiency of SPP1-siRNA was investigated by Western blot assay. GAPDH was used as a loading control (n=3). **B**) Representative graph of migration and invasion assay of ID8 cells indirectly cocultured with RAW264.7 macrophages that were transiently transfected with SPP1-specific siRNA or control siRNA (n=3). The scale bars represent 100 μm. **C, D**) Bar graph showing the statistical analysis of B. **E**) CCK8 kit to detect the proliferation curve of ID8 after co-culture with supernatant from RAW264.7 knocked down with SPP1 siRNA or control (n=3). **F**) TRITC-dextran tracer fluorescence from RAW264.7 and endothelial cells (C166) coculture systems in which macrophages transiently transfected with SPP1-specific siRNA or control siRNA (n=3). Data are mean ± SEM. **P < 0.01, ***P < 0.001 and ****P < 0.0001. One-way ANOVA analysis for C, D and F. Two-way ANOVA analysis for E.


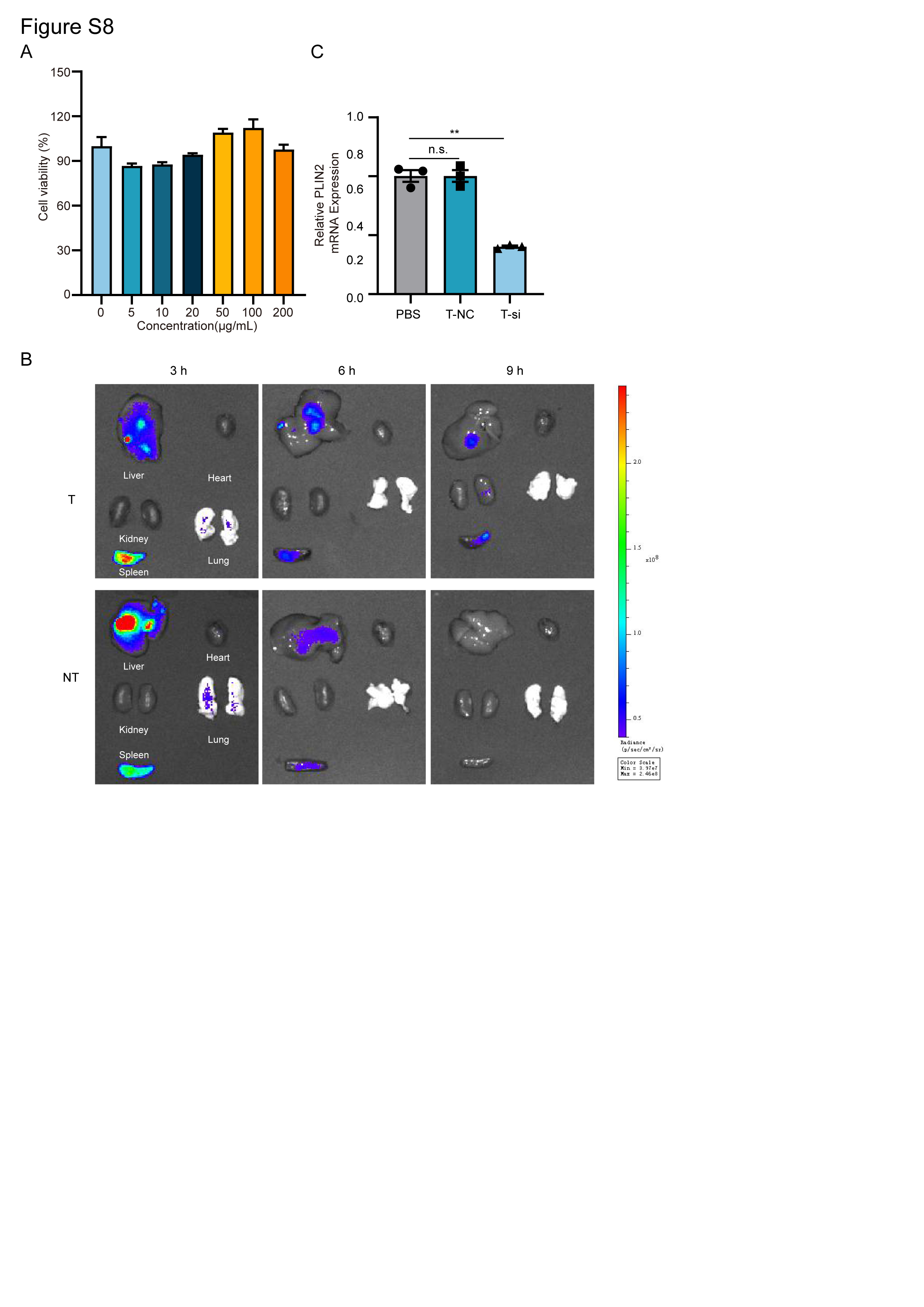


**Figure S8. Therapy of liposome targeting PLIN2.**

**A**) Cell toxicity of liposome on BMDM cells for 72 hours (n=3). **B**) qPCR analysis of PLIN2 knockdown effect by liposome in murine models (n=3). **C**) Uptake of liposome were validated in murine models and representative pictures were shown. Data are presented as mean ± SEM. **P < 0.01; n.s. not significant. One-way ANOVA analysis for B.
